# Transcriptome and metabolome analyses reveal molecular mechanisms of anthocyanin-related leaf color variation in poplar (*Populus deltoides*) cultivars

**DOI:** 10.3389/fpls.2023.1103468

**Published:** 2023-02-24

**Authors:** Xu Qian Peng, Yu Jie Ai, Yu Ting Pu, Xiao Jing Wang, Yu Hang Li, Zhong Wang, Wei Bing Zhuang, Bing Jun Yu, Zhi Qi Zhu

**Affiliations:** ^1^ College of Tea Science, Guizhou University, Guiyang, China; ^2^ Jiangsu Key Laboratory for the Research and Utilization of Plant Resources, Institute of Botany, Jiangsu Province and Chinese Academy of Sciences (Nanjing Botanical Garden Mem. Sun Yat-Sen), Nanjing, China; ^3^ Laboratory of Plant Stress Biology, College of Life Sciences, Nanjing Agricultural University, Nanjing, China; ^4^ Laizhou, Ornamental Research Center, Hongshun Plum Planting Technology Co., Ltd, Yantai, China

**Keywords:** anthocyanin, poplar, RNA-seq, metabolome profiling, correlation analysis

## Abstract

**Introduction:**

Colored-leaf plants are increasingly popular for their aesthetic, ecological, and social value, which are important materials for research on the regulation of plant pigments. However, anthocyanin components and the molecular mechanisms of anthocyanin biosynthesis in colored-leaf poplar remain unclear. Consequently, an integrative analysis of transcriptome and metabolome is performed to identify the key metabolic pathways and key genes, which could contribute to the molecular mechanism of anthocyanin biosynthesis in the colored-leaf cultivars poplar.

**Methods:**

In this study, integrated metabolite and transcriptome analysis was performed to explore the anthocyanin composition and the specific regulatory network of anthocyanin biosynthesis in the purple leaves of the cultivars ‘Quanhong’ (QHP) and ‘Zhongshanyuan’ (ZSY). Correlation analysis between RNA-seq data and metabolite profiles were also performed to explore the candidate genes associated with anthocyanin biosynthesis. R2R3-MYB and bHLH TFs with differential expression levels were used to perform a correlation analysis with differentially accumulated anthocyanins.

**Results and discussion:**

A total of 39 anthocyanin compounds were detected by LC-MS/MS analysis. Twelve cyanidins, seven pelargonidins, five delphinidins, and five procyanidins were identified as the major anthocyanin compounds, which were differentially accumulated in purple leaves of QHP and ZSY. The major genes associated with anthocyanin biosynthesis, including structural genes and transcription factors, were differentially expressed in purple leaves of QHP and ZSY through RNA-sequencing (RNA-seq) data analysis, which was consistent with quantitative real-time PCR analysis results. Correlation analysis between RNA-seq data and metabolite profiles showed that the expression patterns of certain differentially expressed genes in the anthocyanin biosynthesis pathway were strongly correlated with the differential accumulation of anthocyanins. One R2R3-MYB subfamily member in the SG5 subgroup, Podel.04G021100, showed a similar expression pattern to some structural genes. This gene was strongly correlated with 16 anthocyanin compounds, indicating that Podel.04G021100 might be involved in the regulation of anthocyanin biosynthesis. These results contribute to a systematic and comprehensive understanding of anthocyanin accumulation and to the molecular mechanisms of anthocyanin biosynthesis in QHP and ZSY.

## 1 Introduction

Colored-leaf plants are naturally occurring, and the leaves of cultivated species differ in color from the natural green in the entire growing season. Leaf color is an important ornamental trait of cultivated colored-leaf plants. The change in leaf color is strongly associated with the synthesis and accumulation of anthocyanins in leaves (Gao et al., 2021; Li et al., 2021). Anthocyanins, a subclass of flavonoids, perform many biological functions in higher plants, including responsibility for a variety of colors to act as insect and animal attractants (Landi et al., 2015; Liu et al., 2018; Liang and He, 2018). Anthocyanins also act as sunlight attenuators, antioxidants, mediators of reactive oxygen species-induced signaling cascades, chelating agents for heavy metals, and delay leaf senescence in plants (Naing and Kim, 2021; Zheng et al., 2021). Recent studies have shown that anthocyanins could confer tolerance to low temperature, drought and high salinity stress in plants (Zheng et al., 2019; Cirillo et al., 2021; Naing and Kim, 2021). Moreover, anthocyanins are important for the beneficial human-health effects associated with anti-inflammatory, chemopreventive, and antioxidant properties (Dharmawansa et al., 2020; Sirilun et al., 2022). Anthocyanins are responsible for orange, red, magenta, violet and blue colors. The biosynthetic pathway leading to anthocyanin pigment accumulation in flowers or fruit has been well characterized, and the structural genes encoding relevant enzymes and transcriptional factors (TFs) have been isolated (Xie et al., 2011; Vimolmangkang et al., 2013; Chaves-Silva et al., 2018; Yan et al., 2021). The structural genes involved in anthocyanin biosynthesis primarily include phenylalanine ammonia-lyase (*PAL*), chalcone synthase (*CHS*), chalcone isomerase (*CHI*), flavanone 3-hydroxylase (*F3H*), dihydroflavonol 4-reductase (*DFR*), anthocyanidin synthase (*ANS*), and flavonoid 3-*O*-glucosyltransferase (*UFGT*) (Xie et al., 2011; Vimolmangkang et al., 2013; Chaves-Silva et al., 2018; Yan et al., 2021). These genes have been well characterized in many species (Xie et al., 2011; Vimolmangkang et al., 2013; Chaves-Silva et al., 2018; Yan et al., 2021). In addition to glycosylation, modifications such as methylation and acylation contribute to the structural diversity of anthocyanins (Provenzano et al., 2014; Shi et al., 2014; Enaru et al., 2021).

Transcription factors play critical roles in the regulation of plant organ pigmentation (Ambawat et al., 2013; Cheng et al., 2014; Sagawa et al., 2016; Liu et al., 2022). Anthocyanin accumulation is highly regulated by TFs, in particular, by MYB, bHLH, and WD40 proteins (Lin-Wang et al., 2010; Rouholamin et al., 2015; Chen et al., 2019). The MYB TFs have the following two regions: (1) a conserved N-terminal domain and (2) a diverse C-terminal modulator region, responsible for the regulatory activity of the protein. The MYB TF family is subdivided into four subfamilies: 1R-, R2R3-, 3R-, and 4R-MYB proteins (Tombuloglu et al., 2013). Among these subfamilies, R2R3-MYB TFs activate the flavonoid biosynthetic pathway (Lin-Wang et al., 2010). To date, several MYB TFs involved in anthocyanin biosynthesis have been identified in poplar, including PtrMYB116, PtrMYB117, PtrMYB118, PtrMYB119, PtrMYB120, PtrMYB57, and PdMYB118 (Zhou et al., 2018; Wang et al., 2019). The expression of genes associated with anthocyanin biosynthesis is regulated by the MYB-bHLH-WD40 (MBW) protein complex. PtrMYB57 interacts with bHLH131 (a bHLH TF) and PtrTTG1 (a WDR TF) to form the MBW complex and binds to flavonoid gene promoters, which inhibits the biosynthesis of anthocyanin and proanthocyanidins in poplar (Wan et al., 2017). PdMYB118 directly interacts with PdTT8 (a bHLH TF) to regulate wound-induced anthocyanin biosynthesis in *Populus deltoides* (Wang et al., 2020). In addition, several other families of TFs (bZIP, NAC, MADS box, Dof, and WRKY) have been described as regulators of anthocyanin biosynthesis in plants (Riechmann and Ratcliffe, 2000; Sun et al., 2017).

Integration of transcriptome and metabolome analyses not only enable the exploration of new functional genes and metabolites, but also reveal complex molecular mechanisms of secondary metabolite biosynthesis in plants (Carreno-Quintero et al., 2013; Delfin et al., 2019). For example, based on transcriptomics and metabolomics, flavonoid–anthocyanin biosynthetic genes were shown to exhibit differential expression patterns between purple- and green-skinned fruit of *Ficus carica* (Wang et al., 2017). Similarly, accumulation of malvidin 3-*O*-glucoside and delphinidin 3-*O*-glucoside was correlated with reddening of the peel of jujube (*Ziziphus jujuba*) fruit and increased the expression levels of three *UFGT* genes (Liu et al., 2022). Recently, a combined analysis of the fruit metabolome and transcriptome analysis revealed that six candidate genes (*AaF3H*, *AaLDOX*, *AaUFGT*, *AaMYB*, *AabHLH*, and *AaHB2*) and seven flavonoid compounds were closely associated with the pigmentation of red- and green-fleshed cultivars of *Actinidia arguta* (Yu et al., 2020). The flavonoid components and the corresponding molecular mechanisms of colored-leaf cultivars of *P. deltoides* have been explored using transcriptome, metabolome, and proteome analyses (Chen et al., 2021; Tian et al., 2021a). Thus, an integrated metabolome and transcriptome analysis is an effective approach for exploration of anthocyanin components and the molecular mechanisms of anthocyanin biosynthesis in purple leaves of the *P. deltoides* cultivars ‘Quanhong’ (QHP) and ‘Zhongshanyuan’ (ZSY).

Poplar is widely distributed throughout most subtropical regions of the world. The trees exhibit rapid growth and strong stress resistance, and are commonly used for timber, pulp, and paper production (Bal, 2016; Zhuang et al., 2018; Zhuang et al., 2019). In recent years, several types of colored-leaf poplars have been cultivated from a green-leaf cultivar (‘L2025’) (Wang et al., 2022), such as ‘Zhonghong’ (Zhuang et al., 2018), ‘Quanhong’ (QHP) (Zhang et al., 2014; Zhang et al., 2016), ‘Jinhong’ (Zhuang et al., 2018), ‘Caihong’ (Zhuang et al., 2018), and ‘Zhongshanyuan’ (ZSY) (Zhuang et al., 2021a). Recently, ZSY, a valuable colored-leaf cultivar with green and purple leaves, has been used to reveal the molecular regulatory mechanism of anthocyanin biosynthesis (Zhuang et al., 2021b). However, most research on anthocyanin biosynthesis has focused on fruit color (e.g., Rahim et al., 2014; Honda and Moriya, 2018; Zong et al., 2019) and petal color (e.g., Jiang et al., 2014; Han et al., 2020), and anthocyanin biosynthesis in colored-leaf plants has been less thoroughly researched. In the present study, we performed metabolome and transcriptome analyses of the purple leaves of QHP and ZSY. Differentially expressed genes (DEGs), including structural genes and TFs involved in the regulation of anthocyanin biosynthesis, were identified, and the expression patterns of selected DEGs were verified using quantitative real-time PCR (qRT-PCR) analysis. Correlation analysis between RNA-sequencing (RNA-seq) data and metabolite profiling revealed that certain structural genes and TFs were strongly correlated with quantitative changes in anthocyanins. The data from this study improve understanding of anthocyanin biosynthesis in purple leaves of QHP and ZSY at the metabolic and molecular levels, and provide valuable information for the development of new colored-leaf cultivars of poplar. Our study aimed to explore the candidate genes associated with anthocyanin biosynthesis in colored-leaf poplar, which also could contribute to the understanding of molecular mechanisms in anthocyanin biosynthesis of colored-leaf poplar, and the cultivation of new varieties in colored-leaf plants.

## 2 Materials and methods

### 2.1 Plant material

QHP with bright purple leaves, L2025 with green leaves, and ZSY with bright purple and green leaves were planted in the experimental field of Nanjing Botanical Garden, Memorial Sun Yat-Sen (32°3′N, 118°49′E). The fourth and fifth fully expanded mature leaves were collected from two-year-old seedlings of QHP (P) and L2025 (G), respectively. Fully expanded purple leaves (F_P) and green leaves (F_G) from two-year-old ZSY seedlings were also harvested. The leaf samples were immediately frozen in liquid nitrogen and stored at −80°C until use for the determination of chlorophyll, carotenoid, and anthocyanin contents, metabolite detection, RNA-seq, and qRT-PCR analyses. Three independent biological replicates were used for each experiment.

### 2.2 Chlorophyll, carotenoid, and anthocyanin content analysis

The contents of chlorophyll, carotenoid, and anthocyanin of the QHP, ZSY, and L2025 leaves were determined based on a previously described method (Tian et al., 2021b). Fresh leaves were used to measure total chlorophyll content based on the method described by Wu et al. (2020). Leaf tissue (0.1 g) was ground into powder and extracted in 5 mL of 95% ethanol at 50°C for 2 h. The mixture was vortexed and centrifuged at 5,000 rpm for 5 min. The absorbance of the supernatant was measured at 470, 649, and 665 nm using an ultraviolet–visible spectrophotometer (Wu et al., 2020; Zhuang et al., 2021b). Fresh leaves were used to determine the carotenoid content in accordance with the method used by (Gao et al., 2021). Approximately 100 mg of fresh leaf tissue were cut into pieces with scissors, and extracted in 10 mL of 1% (v/v) HCl–ethanol at 60°C for 30 min. The mixture was vortexed and centrifuged at 13,000 × *g* for 5 min. The absorbance of the supernatant was measured at 530, 620, and 650 nm using an ultraviolet-visible spectrophotometer (Gao et al., 2021).

### 2.3 Estimation of total anthocyanin content

The total anthocyanin content was determined in accordance with the method of Xu et al. (2017) using functional leaves (the third to fifth leaves from the main branches) of two-year-old seedlings of QHP, ZSY, and L2025.

### 2.4 Metabolite profile analysis

Metabolite profiling of the extracted leaf samples was performed using a liquid chromatography–tandem mass spectrometry (LC-MS/MS) (Ma et al., 2006). Chromatographic separation of anthocyanin metabolites was performed on an ACQUITY UPLC HSS T3 column (100 mm × 2.1 mm, 1.8 µm), using a gradient elution of 0.1% formic acid (Solution A) with acetonitrile (Solution B) at a flow rate of 0.2 mL/min and temperature of 40°C. The elution profile was set as follows: 80:20 VA/VB at 0 min, 80:20 VA/VB at 1 min, 25:75 VA/VB at 3 min, 25:75 VA/VB at 4 min, 80:20 VA/VB at 4.05 min, 80:20 VA/VB at 6.5 min, and 80:20 VA/VB at 7.5 min. The injection volume was 10 μL.

A quadrupole-time-of-flight (TOF) tandem mass spectrometer was operated in both positive and negative ion modes. The optimized parameters were as follows: the ion source temperature 500°C; ion spray voltage 5.5 kV (positive), −4.5 kV (negative); ion sources gas1, ion sources gas2, and curtain gas were set at 50, 50, and 35 psi, respectively. The collision gas and ion spray voltage were set to medium and 4.5 kV, respectively. Data acquisition and analysis were performed using Analyst Software (Sciex, Foster City, CA, USA).

### 2.5 Qualitative and quantitative analysis of metabolites

Data preprocessing was performed using Progenesis QI software to obtain *m/z* values, retention time (RT), and peak intensity. Metabolites were identified by searching and comparing the *m/z* values, RT, and fragmentation patterns with standards in the Human Metabolome Database (HMDB; http://www.hmdb.ca/) and Metlin database (https://metlin.scripps.edu/). The variable importance of projection (VIP) score of the orthogonal projections to latent structures–discriminant analysis model was used to detect global metabolic changes among comparable groups. Metabolites with a *t*-test adjusted *p*-value (*p*_adj_) ≤ 0.05 and VIP ≥ 1 were defined as differential metabolites. The Bioconductor R package from on the Majorbio Cloud Platform (https://cloud.majorbio.com) was used to perform multivariate statistical analysis. An unsupervised method was used to perform a principal component analysis (PCA).

### 2.6 RNA extraction, library construction, and sequencing

Total RNA was extracted from the frozen leaf samples of each poplar cultivar (QHP, ZSY, and L2025) using the EASYspin Plus Plant RNA Kit (Aidlab, Beijing, China). The extracted RNA was treated with DNase I (TaKaRa) to remove the contaminant DNA. The Dynabeads™ mRNA Purification Kit (Invitrogen) was used for the purification of mRNA from total RNA. The purified mRNAs were cleaved into small fragments and reverse-transcribed into first-strand cDNAs with random hexamer primers. The double-stranded cDNAs were synthesized using the NEBNext Ultra RNA Library Prep Kit (NEB). Adaptors were added to the double-stranded cDNAs, which were further fragmented and enriched by PCR to construct the final cDNA libraries for Illumina paired-end sequencing using an Illumina NovaSeq 600 platform.

### 2.7 Analysis of RNA-seq data

After trimming the adapter sequences and removing low quality sequences, the clean reads were mapped to the *P. deltoides* reference genome (https://www.ncbi.nlm.nih.gov/data-hub/genome/GCA_015852605.2/) and assembled using Hisat2 and StringTie, respectively. The obtained transcripts were annotated using eight databases (Nr, Nt, GO, Swiss-Prot, COG, KOG, Pfam, and KEGG). Gene expression levels were calculated and normalized using fragments per kilobase of transcript per million mapped fragments (FPKM). The DEGs between two groups were analyzed using the DESeq R package. A false discovery rate (*p*_adj_) ≤ 0.05 and |log_2_(fold change)| ≥ 1 were used as the criteria to identify significant DEGs (Ali and Bhaskar, 2016). In addition, gene ontology (GO) and Kyoto Encyclopedia of Genes and Genomes (KEGG) analyses of DEGs were performed using the DAVID and KOBAS online tools, respectively. The RNA-seq dataset is available from the Gene Expression Omnibus (accession no. GSE216754).

### 2.8 Correlation analysis of metabolite and transcriptome profiling

Pearson’s correlation analysis was conducted by calculating the correlation coefficient between anthocyanin content and the expression of DEGs enriched in the flavonoid–anthocyanin biosynthesis pathway (ko00941 and ko00942). Furthermore, R2R3-MYB and bHLH TFs with differential expression levels were used to perform a correlation analysis with differentially accumulated anthocyanins. Interaction networks between DEGs and differentially accumulated anthocyanins were visualized using Cytoscape 2.8.2 (Cho et al., 2016).

### 2.9 qRT-PCR analysis

The differential expression patterns of selected structural genes and TFs in the anthocyanin biosynthesis pathway, as detected from the RNA-seq data, were investigated using qRT-PCR (Kodama et al., 2018). The *Actin* gene was used to normalize the gene expression levels in all groups (Wang et al., 2015). The relative expression level for each gene was calculated using the 2^−ΔΔCt^ method. Three technical replicates and three biological replicates were analyzed for each sample. All primers used in this study are listed in **Supplementary Table 1**.

### 2.10 Statistical analysis

Heatmap analysis, PCA, and multiple-testing of metabolome and transcriptome data were conducted using R software. Results are expressed as the mean ± standard deviation (SD). The calculated *p*-value was calibrated by false discovery rate (FDR) correction.

## 3 Results

### 3.1 Pigment constituents in leaves of QHP and ZSY

**Figure 1:**
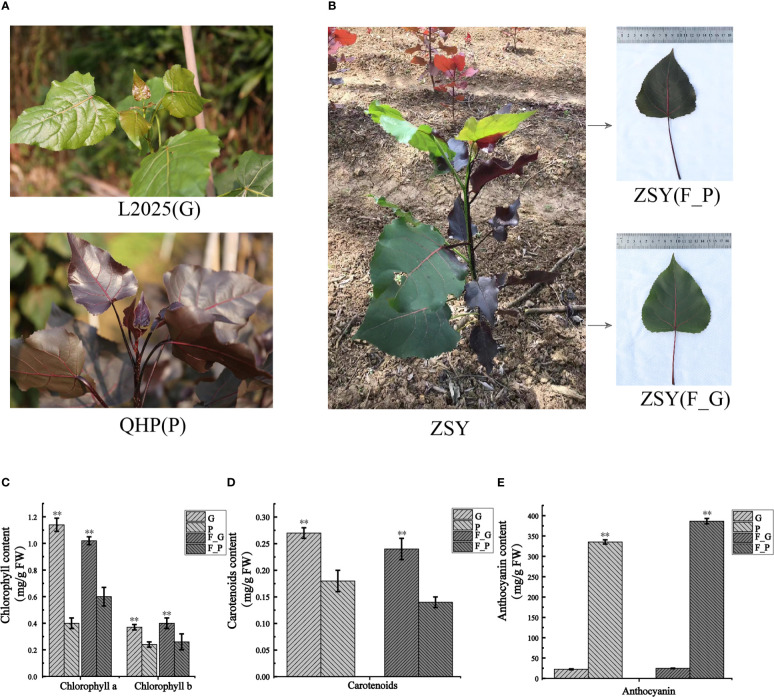
Morphological phenotype and pigment constituents of Populus deltoides ‘Quanhong’ (QHP), ‘Zhongshanyuan’ (ZSY), and ‘L2025’. **(A)** Morphological phenotype of QHP and L2025; P, purple leaves of QHP; G, green leaves of L2025. **(B)** Morphological phenotype of ZSY; F_P and F_G, purple leaves and green leaves, respectively. **(C)** Chlorophyll content in leaves of QHP, ZSY, and L2025. **(D)** Carotenoid content in the leaves of QHP, ZSY, and L2025. **(E)** Anthocyanin content in leaves of QHP, ZSY, and L2025. Data are presented as the mean ± SD (N = 3). **p < 0.001 (Student’s t-test).

To detect the pigment constituents in purple leaves of QHP and ZSY, the contents of total chlorophyll (Chl-a and Chl-b), carotenoids, and anthocyanins in leaves of L2025, QHP, and ZSY were measured (**Figures 1A, B**). The Chl-a, Chl-b, and carotenoid contents of L2025 leaves (G) were 2.85, 1.54, and 1.50 times those of QHP leaves (P), respectively, and the anthocyanin content of QHP leaves (P) was 14.82 times that of the L2025 leaves (G) (**Figures 1C–E**). The Chl-a, Chl-b, and carotenoid contents of ZSY green leaves (F_G) were 1.70, 1.54, and 1.71 times those of ZSY purple leaves (F_P), whereas the anthocyanin content of ZSY purple leaves was 15.69 times that of ZSY green leaves (**Figures 1C–E** and **Supplementary Table 2**). These results indicated that the purple color of the leaves may have been caused by anthocyanin accumulation.

### 3.2 Anthocyanin contents in leaves of different colors

**Figure 2:**
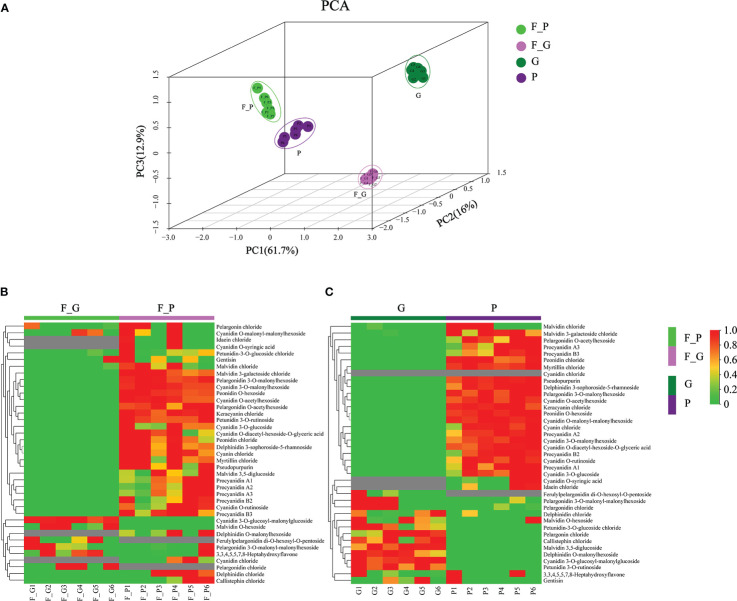
Principal component analysis (PCA) and differential accumulation of anthocyanin metabolites in leaves of *Populus deltoides* ‘Quanhong’ (QHP) and ‘Zhongshanyuan’ (ZSY). **(A)** PCA scatterplot of the contents of anthocyanin compounds. **(B, C)** represent heat maps of the differential accumulation of anthocyanin metabolites in the F_P vs F_G and P vs G comparison groups, respectively. P, purple leaves of QHP; G, green leaves of L2025; F_P and F_G, purple leaves and green leaves of ZSY, respectively. Red and green shading represent high and low contents of anthocyanins, respectively.

To further explore the anthocyanin components and the corresponding molecular mechanisms of anthocyanin biosynthesis in purple leaves of QHP and ZSY, the same leaf samples, comprising the green leaves of L2025 and ZSY (G and F_G), and the purple leaves of QHP and ZSY (P and F_P), were used for metabolite and transcriptome analysis. Metabolite profiling of the leaves (G, P, F_G, and F_P) was performed using LC-MS/MS analysis. A total of 39 anthocyanin metabolites were identified: 12 cyanidins, 7 pelargonidins, 5 delphindins, 5 procyanidins, 4 malvidins, 2 petunidins, 2 peonidins, 1 pseudopurpurin, and 1 gentisin (**Table 1**). The PCA showed clear separation of the sample groups. Among the leaf samples, 90.6% of the total variance was explained by PC1 (61.7%), PC2 (16%), and PC3 (12.9%) (**Figure 2A**). The PCA revealed lower variability among the biological replicates. To identify anthocyanin compounds that were differentially accumulated between the purple and green leaves, the anthocyanins from the leaf samples were subjected to comparative analysis according to their relative contents: P *vs*. G and F_P *vs*. F_G. The comparison between F_P and F_G showed that 22 anthocyanins increased, whereas 11 decreased (**Figure 2B**). Between P and G, 24 anthocyanins increased, whereas two decreased (**Figure 2C**). In particular, 22 differentially accumulated anthocyanins, including 20 upregulated anthocyanins (7 cyanidins, 5 procyanidins, 2 delphinidins, 2 pelargonidins, 2 peonidins, 1 malvidin, and 1 pseudopurpurin) and two downregulated anthocyanins (cyanidin and malvidin *O*-hexoside), were detected in both comparisons P vs. G and F_P vs. F_G (**Supplementary Table 3**).

**Table 1:** List of anthocyanins in poplar leaves identified by metabolome analysis.

Type	Name	Q1 [Da]	RT [min]	QC1	QC2	QC3	F_G	F_P	G	P
Cyanidin	Cyanidin 3-*O*-malonylhexoside	535.1	3.739	121900000	134100000	125700000	0.02	0.33	0.07	0.92
	Cyanidin *O*-malonyl-malonylhexoside	621.1	3.663	84270000	84090000	72600000	0.58	0	0.36	0
	Cyanidin chloride	303.1	3.424	78470	75950	73200	0.02	0.64	0.01	0.51
	Cyanidin 3-*O*-glucosyl-malonylglucoside	697.1	3.745	387800	407000	390400	0.01	0.27	0.9	0.37
	Keracyanin chloride	611.1	1.561	62240	61760	59120	0.02	0.54	0.1	0.87
	Cyanidin *O*-syringic acid	465.1	1.555	4195000	4534000	4184000	0	0.2	0	0.76
	Cyanidin *O*-diacetyl-hexoside-*O*-glyceric acid	619.1	3.768	19720000	19680000	19700000	0.02	0.9	0.03	0.6
	Cyanidin *O*-acetylhexoside	489.1	3.739	111300000	105700000	98630000	0.06	0.21	0.01	0.86
	Cyanidin *O*-rutinoside	595.17	1.525	83530	97370	93900	0.12	0.24	0.59	0.34
	Cyanidin 3-*O*-glucoside	465.1	1.614	1456000	1521000	1699000	0.03	0.6	0.01	0.5
	Idaein chloride	465.1	1.56	3820000	3825000	3419000	0.02	0.45	0.01	0.82
	Cyanin chloride	627.1	1.37	28220	25150	24270	0.37	0.74	0.79	0.12
Pelargonidin	Pelargonidin 3-*O*-malonylhexoside	519.1	3.795	25910000	28140000	23440000	0.01	0.4	0.01	0.65
	Pelargonidin 3-*O*-malonyl-malonylhexoside	605.1	1.129	42990	47810	34960	0.01	0.44	0	0.89
	Pelargonin chloride	611.1	1.544	94960	102600	91110	0.01	0.38	0	0.78
	Pelargonidin chloride	287	3.773	7090	5343	3217	0.04	0.12	0.72	0.18
	Pelargonidin *O*-acetylhexoside	473.1	3.803	5696000	5660000	5964000	0.85	0	0.31	0.26
	Callistephin chloride	449.1	1.647	564000	573700	612600	0.05	0.41	0.1	0.79
	Ferulylpelargonidin di-*O*-hexosyl-*O*-pentosid	919	3.599	3531	3536	2248	0.03	0.05	0.82	0.35
Peonidin	Peonidin chloride	317.1	3.812	77660	75180	67660	0	0.4	0	0
	Peonidin *O*-hexoside	463.123	3.646	136600000	141200000	151200000	0	0.21	0	0.74
Malvidin	Malvidin 3-galactoside chloride	509.1	1.719	37630	36760	37880	0	0.89	0.08	0.52
	Malvidin *O*-hexoside	493.1	1.734	10240	7730	6349	0.83	0.74	0.12	0.06
	Malvidin 3,5-diglucoside	655.2	1.721	192000	187300	164300	0.59	0.29	0.5	0.2
	Malvidin chloride	347.1	3.812	5987	7555	5333	0.03	0.59	0.02	0.69
Delphinidin	Delphinidin chloride	319	4.797	2619	3135	3109	0	0.08	0.76	0.02
	3,3’,4’,5,5’,7,8-Heptahydroxyflavone	366.892	1.018	1230	2130	2333	0.01	0.2	0.03	0.91
	Myrtillin chloride	481.1	1.483	73020	72930	76280	0.02	0.89	0	0.89
	Delphinidin *O*-malonylhexoside	551.1	3.661	1231000	1359000	1394000	0.07	0.92	0.01	0.66
	Delphinidin 3-sophoroside-5-rhamnoside	773	3.547	766600	767800	842300	0.02	0.65	0.16	0.94
Petunidin	Petunidin 3-*O*-rutinoside	625.3	3.538	76650000	73980000	79850000	0.1	0.77	0.01	0.58
	Petunidin-3-*O*-glucoside chloride	495.1	1.576	5880	6644	8018	0.09	0.36	0.43	0.68
	Gentisin	290.892	4.514	39910	22200	21390	0.11	0.13	0.02	0.96
	Pseudopurpurin	332.892	3.496	41060	49740	47530	0.53	0.14	0.94	0.07
Procyanidin	Procyanidin A3	577.1	1.996	1135000	1119000	1206000	0.09	0.35	0.71	0.39
	Procyanidin A1	577	1.9	543300	595700	532500	0.01	0.86	0.21	0.47
	Procyanidin A2	577	2.001	514600	509300	510200	0.67	0.43	0.32	0.28
	Procyanidin B2	577.135	1.895	4912000	4514000	4709000	0.46	0.58	0.42	0.36
	Procyanidin B3	577.1	1.908	4619000	4819000	5044000	0.04	0.84	0.24	0.44

### 3.3 Correlation analysis of metabolites

**Figure 3:**
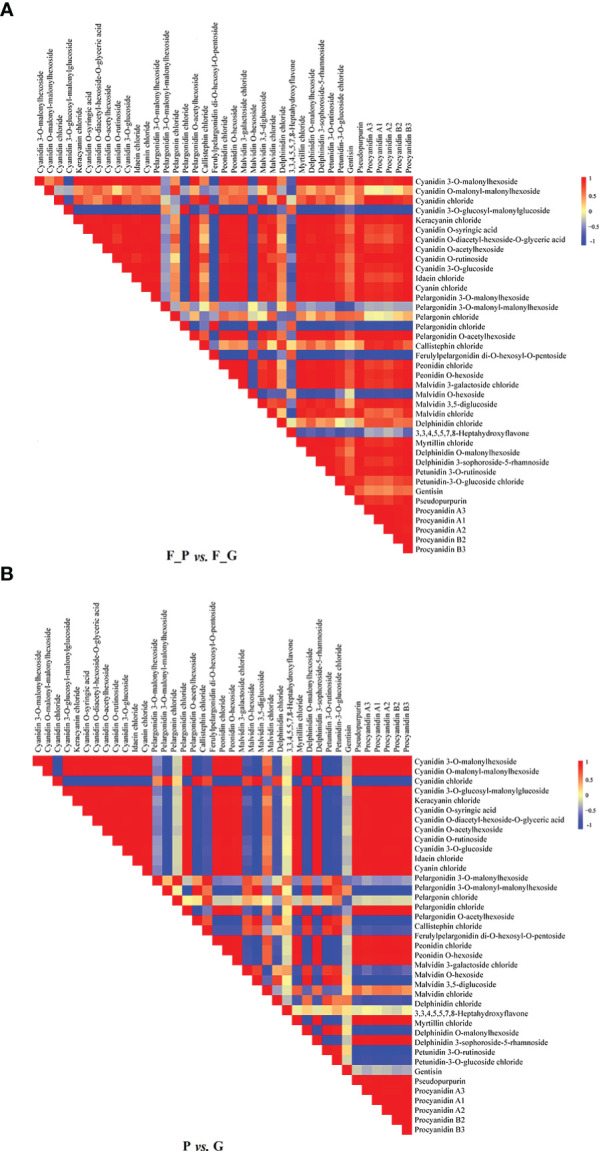
Visualization of metabolite–metabolite correlations. **(A)** Heat map of pairwise correlations between metabolites identified in the F_P vs F_G comparison group. **(B)** Heat map of pairwise correlations between metabolites identified in the P vs G comparison group. Each square indicates a given *r* value resulting from a Pearson correlation analysis in a false color scale (red and blue indicate positive and negative correlations, respectively). The self-comparisons are indicated in white.

Correlation heat maps were constructed from the differential anthocyanin metabolites detected from the comparisons P vs. G and F_P vs. F_G (**Figure 3**). Of 741 pairwise correlations from 39 anthocyanin metabolites, 367 positive (yellow to red) and 95 negative (yellow to blue) correlations (altogether approximately 62.3% of the total) were observed for P vs. G (**Figure 3A**), and 297 positive and 142 negative correlations (altogether approximately 69.2% of the total) were observed for F_P vs. F_G (**Figure 3B**). Significant changes in metabolite–metabolite correlations (both positive and negative) were observed between P vs. G and F_P vs. F_G. This in turn showed that adjustments in the branches of the anthocyanin metabolic pathway occurred during the development of purple leaves in QHP and ZSY. In P vs. G, 31.51% of 367 positive correlations and 47.91% of 95 negative correlations were for procyanidins, and cyanidins–other anthocyanins correlations, respectively. 33.67% of 297 positive correlations and 43.26% of 142 negative correlations were for procyanidins, and cyanidins–other anthocyanins correlations, respectively, that were observed in F_P vs. F_G (**Supplementary Table 4**). These results indicated that procyanidins and cyanidins were the main contributors to the differences in accumulated anthocyanin metabolites between QHP and ZSY.

### 3.4 Transcriptome analysis of green and purple leaves

To explore the molecular regulatory mechanisms of anthocyanin biosynthesis during the development of purple leaves of QHP and ZSY, 12 cDNA libraries were constructed from the same samples used for metabolite analysis to enable high-throughput RNA-seq (**Supplementary Table 5**). In total, 535,787,836 raw reads in the range of 42,021,546–46,112,070 and 510,395,564 clean reads in the range of 41,235,222–44,584,822 were obtained for each library (**Supplementary Table 6**). The Q30 percentage and average GC content percentages were more than 97% and 43.93% for the libraries, respectively. Overall, 80.23%–92.71% of the clean reads were mapped to the genome of *P. deltoides* for each library. Thus, 32,819, 32,704, 34,047, 32,331, 32,321, 32,337, 34,290, 34,098, 33,214, 33,014, 33,300, and 32,998 genes for each library were identified. These results demonstrated that the RNA-seq data were of high quality and suitable for subsequent analyses.

### 3.5 Differentially expressed genes

**Figure 4:**
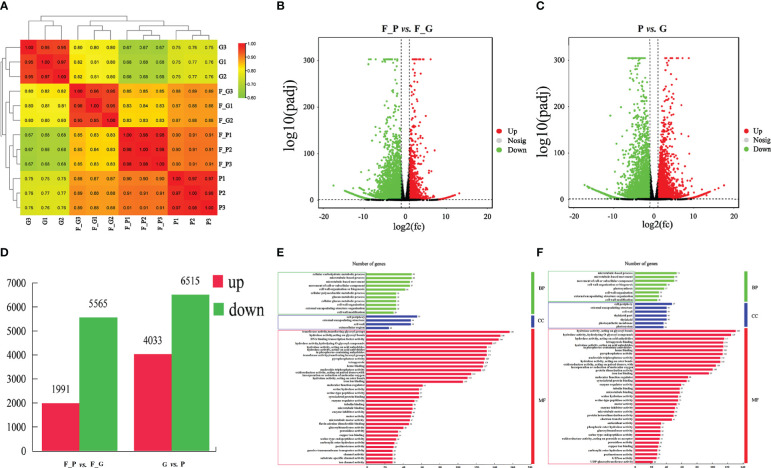
Identification and gene ontology enrichment of differentially expressed genes (DEGs) identified in leaves of *Populus deltoides* ‘Quanhong’ (QHP) and ‘Zhongshanyuan’ (ZSY). **(A)** Correlation of all samples. A correlation analysis between the samples was performed to estimate biological duplication among samples within a group. **(B)** Volcano plot of DEGs between F_P and F_G. **(C)** Volcano plot of DEGs between P and G. The two vertical dotted lines are twice the difference threshold, and the horizontal dotted line represents a *p*-value of 0.05. Red dots indicate the upregulated genes in this group, blue dots indicate the downregulated genes in this group, and black dots indicate the non-significantly differentially expressed genes. **(D)** Number of DEGs identified in F_P vs F_G and P vs G. **(E)** Gene ontology enrichment of DEGs in F_P vs F_G. **(F)** Gene ontology enrichment of DEGs in P vs G.

To identify the DEGs between the purple and green leaves of QHP, L2025, and ZSY, the correlation coefficients (*r*) between the purple and green leaves for the repeated samples of QHP, L2025, and ZSY were computed based on the FPKM value of all transcripts in each sample. The *r* values between the biological replicates of the four poplar leaf samples were all greater than 0.9 (**Figure 4A**). The samples were scattered among the four different groups and clustered within the same group, which indicated that the biological replicates could be further used for the detection of DEGs (**Figures 4B, C**). Based on pairwise comparisons with fold change ≥ 1 and FDR ≤ 0.05, 8,217 (2,005 upregulated and 6,212 downregulated) and 11,484 (5,041 upregulated and 6,643 downregulated) DEGs were identified in F_G vs. F_P and P vs. G, respectively (**Figure 4D**).

A GO enrichment analysis was performed to explore the biological functions of DEGs using Blast2GO. A total of 2,345 (F_G vs. F_P) and 3,297 (G vs. P) DEGs were clustered into 83 and 69 functional groups, respectively (**Supplementary Table 7**). The highest-ranked significant GO terms in the enrichment analysis for the DEGs in F_P vs. F_G and P vs. G were classified into three GO categories: 11 and 11 in the biological process category, 4 and 7 in the cellular component category, and 35 and 32 in the molecular function category, respectively (**Figures 4E, F**). Among biological processes, the most enriched terms were microtubule-based movement, microtubule-based process, and cellular carbohydrate metabolic process. In the cellular component category, the cell wall, external encapsulating structure, and cell periphery were the most represented GO terms. For the molecular function category, the most enriched terms were hydrolase activity acting on acid anhydrides, hydrolase activity hydrolyzing *O*-glycosyl compounds, and hydrolase activity acting on glycosyl bond catalytic, binding, and transporter activities.

**Figure 5:**
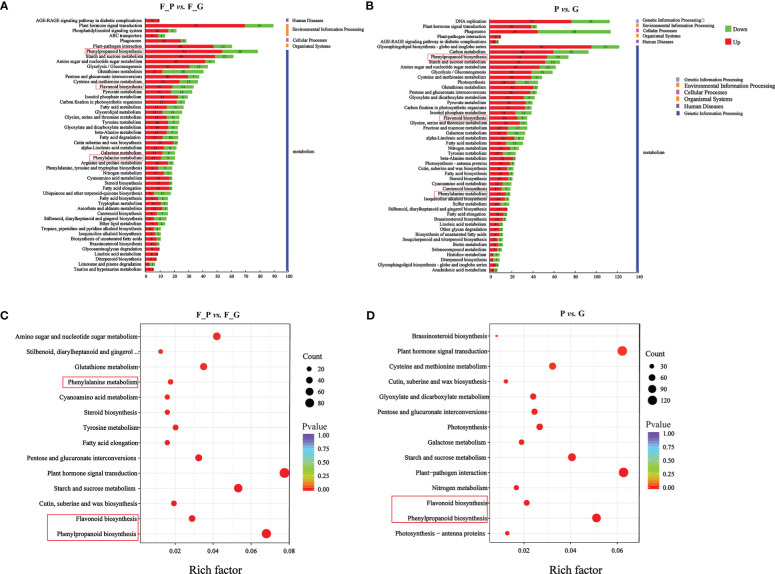
KEGG analysis of the differentially expressed genes (DEGs) in different comparison groups. **(A)** KEGG pathway assignment of the DEGs in F_P *vs.* F_G (top 50 pathways according to the enrichment factor). **(B)** KEGG pathway assignment of the DEGs in P *vs.* G (top 50 pathways according to the enrichment factor). The vertical axis represents the enriched KEGG pathways, and the horizontal axis represents the number of DEGs in each pathway. **(C)** Significantly enriched KEGG pathways (*P* < 0.05) from DEGs in F_P vs F_G. **(D)** Significantly enriched KEGG pathways (*P* < 0.05) from DEGs in P vs G The red frames indicated the phenylpropanoid and flavonoid biosynthesis pathways.

To further explore the DEGs involved in the metabolic pathways, a KEGG pathway analysis was performed. A total of 1,158 (F_P vs. F_G) and 1,818 (P vs. G) DEGs were assigned to 121 KEGG pathways (**Supplementary Table 8**). The 50 highest-ranked significant KEGG pathways were classified into five categories: metabolism, genetic information processing, cellular processes, environmental information processing, and organismal systems. Among these pathways, the five metabolic pathways with the largest number of DEGs were annotated using the KEGG database in two pairwise comparisons: F_P vs. F_G and P vs. G (**Figures 5A, B**). The five metabolic pathways with the largest number of DEGs from F_P vs. F_G were plant hormone signal transduction (pop04075, 89 genes), phenylpropanoid biosynthesis (pop00940, 78 genes), carbon metabolism (pop01200, 69 genes), starch and sucrose metabolism (pop00500, 61 genes), and plant–pathogen interaction (pop04626, 60 genes). For the comparison of P vs. G, plant–pathogen interaction (pop04626, 113 genes), plant hormone signal transduction (pop04075, 112 genes), phenylpropanoid biosynthesis (pop00940, 92 genes), starch and sucrose metabolism (pop00500, 72 genes), and amino sugar and nucleotide sugar metabolism (pop00520, 65 genes) were the most highly enriched pathways. Notably, plant hormone signal transduction (pop04075), phenylpropanoid biosynthesis (pop00940), starch and sucrose metabolism (pop00500), and plant–pathogen interaction (pop04626) were present among the significantly enriched pathways in both F_P vs. F_G and P vs. G (**Figures 5C, D**).

### 3.6 DEGs involved in regulation of anthocyanin biosynthesis in colored leaves

**Figure 6:**
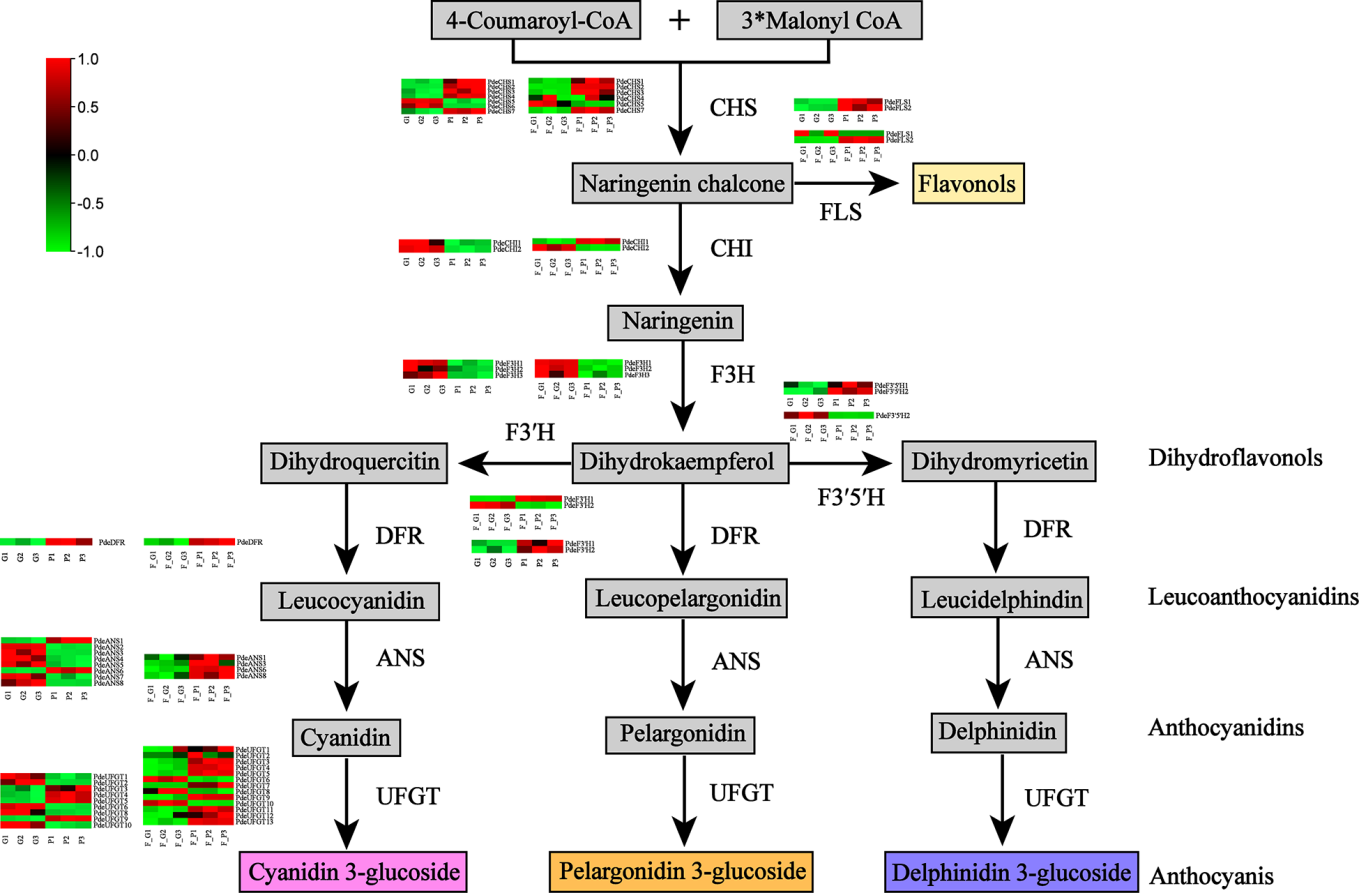
Differentially expressed genes (DEGs) involved in the anthocyanin biosynthesis pathway. Common anthocyanins detected in this study are indicated in gray. Red and green colors indicate a higher or lower expression level of DEGs. Abbreviations: CHS, chalcone synthase; FLS, flavonol synthase; CHI, chalcone isomerase; F3H, flavanone 3-hydroxylase; F3′H, flavonol 3′-hydroxylase; F3′5′H, flavonol 3′5′-hydroxylase; DFR, dihydroflavonol 4-reductase; ANS, anthocyanidin synthase; UFGT, flavonoid-3-*O*-glycosyltransferase.

The DEGs encoding key enzymes involved in anthocyanin biosynthesis were identified based on gene functional annotation and KEGG pathway enrichment analysis (**Figure 6** and **Supplementary Table 9**). As a result, 28 DEGs (four *CHS*, three *FLS*, four *F3H*, two *F3’H*, two *F3’5’H*, one *DFR*, four *ANS*, and eight *UFGT* genes) were detected in F_P *vs*. F_G. Among these genes, three *CHS*, two *FLS*, one *F3’H*, one *F3’5’H*, one *DFR*, two *ANS*, and five *UFGT* genes were significantly upregulated in F_P vs. F_G. The remaining DEGs were downregulated in F_P vs. F_G. Thirty-three DEGs (seven *CHS*, three *FLS*, three *F3H*, three *F3’H*, two *F3’5’H*, one *DFR*, five *ANS*, and nine *UFGT* genes) were detected in P *vs*. G. Among these genes, five *CHS*, three *FLS*, two *F3’H*, one *DFR*, one *ANS*, and four *UFGT* genes were significantly upregulated in P vs. G. The remaining DEGs were downregulated in the P vs. G group. In addition, 11 DEGs, comprising three *CHS* (*PdeCHS1*, *PdeCHS2*, and *PdeCHS7*), two *FLS* (*PdeFLS3* and *PdeFLS4*), one *F3’H* (*PdeF3’H1*), one *DFR* (*PdeDFR*), one *ANS* (*PdeANS3*), and three *UFGT* genes (*PdeUFGT3*, *PdeUFGT4*, and *PdeUFGT5*), were significantly upregulated in F_P *vs*. F_G and P vs. G.

**Figure 7:**
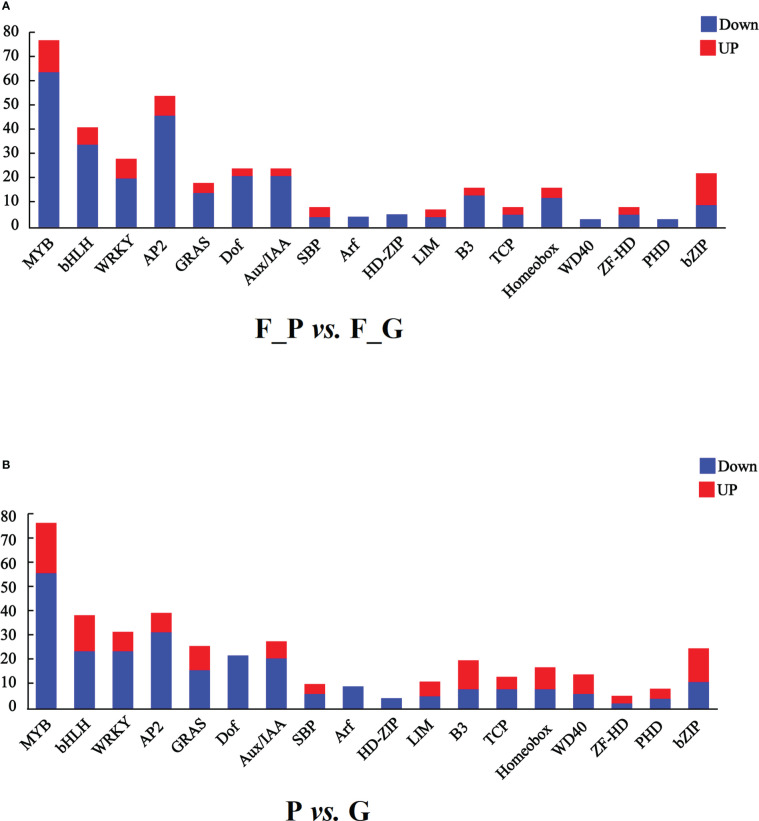
Transcription factors (TFs) differentially expressed in leaves of different poplar cultivars based on the RNA-seq data. **(A)** Number of TFs with a differential expression pattern in F_P vs F_G. **(B)** Number of TFs with differential expression pattern in P vs G. Blue and red columns indicate down- and up-regulated TFs, respectively. P, purple leaves of ‘Quanhong’ (QHP); G, green leaves of ‘L2025’; F_P and F_G, purple leaves and green leaves of ‘Zhongshanyuan’ (ZSY), respectively.

It is well known that MYB, bHLH, and WD40 TFs play important roles in regulating anthocyanin biosynthesis. In addition, AP2/ERF, bZIP, WRKY, and Dof TFs are associated with anthocyanin biosynthesis. In this study, seven TF families were detected as differentially expressed in the RNA-seq data (**Figure 7** and **Supplementary Table 10**). A total of 75 MYB, 39 bHLH, 26 WRKY, 52 AP2, 22 Dof, 20 bZIP, and one WD40 TFs were differentially expressed in the comparison group of F_P vs. F_G. Among these TFs, 11 MYB, 5 bHLH, 6 WRKY, 6 AP2, 11 bZIP, and 1 Dof exhibited similar expression patterns as the structural genes in the anthocyanin biosynthesis pathway, which were increased in purple leaves of ZSY. Seventy-six MYB, 34 bHLH, 20 WRKY, 46 AP2, 21 Dof, 1 bZIP, and 1 WD40 TFs exhibited the opposite expression pattern to most structural genes in the anthocyanin biosynthesis pathway, which decreased in purple leaves of ZSY. Moreover, 76 MYB, 37 bHLH, 12 WD40, 30 WRKY, 38 AP2, 20 Dof, and 23 bZIP TFs were differentially expressed in the comparison group of P vs. G. Among these TFs, 19 MYB, 13 bHLH, 6 WD40, 6 WRKY, 6 AP2, and 12 bZIP TFs showed expression patterns consistent with most anthocyanin biosynthetic structural genes, which increased in the leaves of QHP. In contrast, 57 MYB, 24 bHLH, 24 WRKY, 32 AP2, 20 Dof, 11 bZIP, and 6 WD40 TFs exhibited the opposite expression pattern to most anthocyanin biosynthetic structural genes, which increased in the leaves of L2025. To explore the function of differentially expressed MYB TFs, an unrooted phylogenetic tree of MYBs, including the differentially expressed MYBs in *P. deltoides* and *Arabidopsis* MYBs, was constructed. Two *P. deltoides* MYBs (Podel.06G234300 and Podel.04G021100) belonging to the SG5 subgroup of R2R3-MYB TFs were identified (**Figure S1**).

### 3.7 Validation of expression pattern of DEGs in the anthocyanin biosynthesis pathway by qRT-PCR

**Figure 8:**
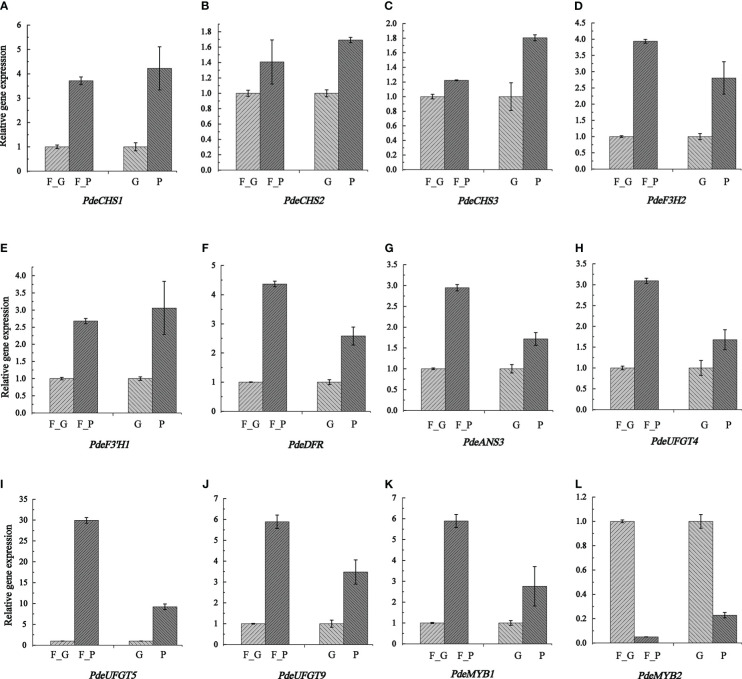
Transcription levels of anthocyanin biosynthesis genes and two anthocyanin regulation MYB TFs as determined by qRT- PCR. Relative expression levels were calculated using *Actin2* as a standard. Three biological replicates and three technical replicates were analyzed for each data point. Data are presented as the mean ± SD (*N* = 3). **(A–L)** indicate the expression levels of *PdeCHS1*, *PdeCHS2*, *PdeCHS3*, *PdeF3H2*, *PdeF3’H1*, *PdeDFR*, *PdeANS3*, *PdeUFGT4*, *PdeUFGT5*, *PdeUFGT9*, *PdeMYB1*, and *PdeMYB2.* F_P and F_G, purple leaves and green leaves of ‘Zhongshanyuan’ (ZSY), respectively; P, purple leaves of ‘Quanhong’ (QHP); G, green leaves of ‘L2025’.

To verify the expression pattern of DEGs involved in anthocyanin biosynthesis, 12 structural genes encoding key enzymes (three *CHS*, one *F3H*, one *F3’H*, one *DFR*, one *CHI*, one *ANS*, and three *UFGT*) and two TFs (two SG5 R2R3-MYB TFs) were subjected to qRT-PCR analysis. The qRT-PCR results showed that the expression pattern of all the selected candidate genes was strongly correlated with the RNA-seq data (**Figure 8** and **Supplementary Table 11**).

### 3.8 Correlation between DEGs and anthocyanin compounds

**Figure 9:**
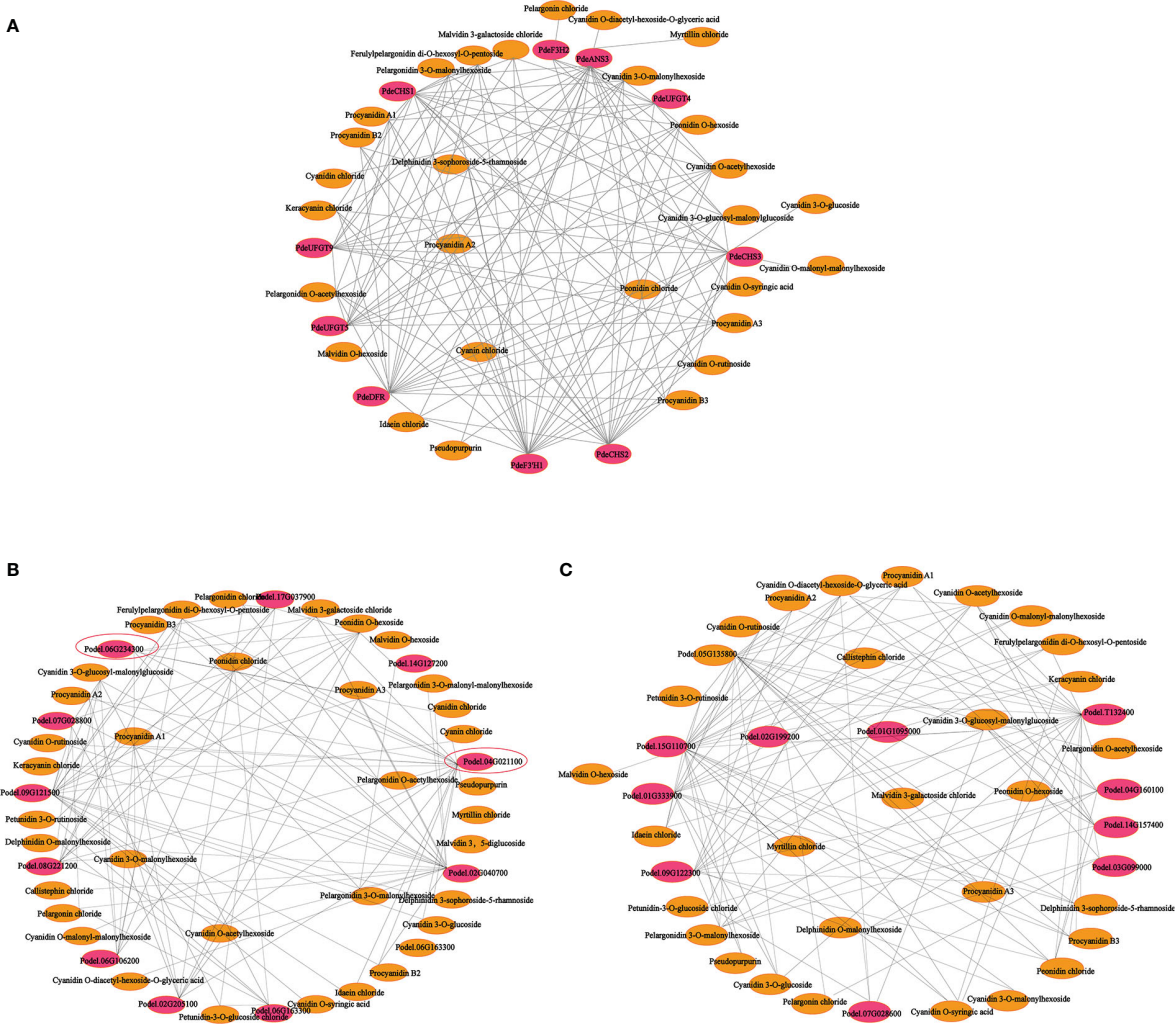
Connection network between anthocyanin biosynthesis-related differentially expressed genes (DEGs) and differentially accumulated anthocyanin metabolites in leaves of different poplar cultivars. **(A)** Connection network between anthocyanin biosynthesis structural genes and anthocyanin metabolites. **(B)** Connection network between MYB transcription factor genes and anthocyanin metabolites. **(C)** Connection network between bHLH transcription factor genes and anthocyanin metabolites. The red ellipsoid represents DEGs; the yellow ellipsoid represents differentially accumulated anthocyanins; the red ellipsoid with the black triangle represents the MYB transcription factor Podel.04G021100.

To further explore the complex regulatory mechanism of anthocyanin biosynthesis in the leaves of colored-leaf poplar, correlation analysis of quantitative changes in anthocyanin content and related DEGs in the leaves of colored-leaf poplar was performed (**Figure 9**). Based on the detected differentially accumulated metabolites and DEGs involved in anthocyanin biosynthesis, 10 structural genes displayed a significant positive correlation (*r* > 0.8) with 28 anthocyanins (**Supplementary Table 12**). The corresponding interaction networks are shown in **Figure 9A**. The 10 structural genes, comprising three *CHS*, one *F3H*, one *F3’H*, one *DFR*, one *ANS*, and three *UFGT*, played a pivotal role in the anthocyanin biosynthesis pathway. The 28 anthocyanins were cyanidin *O*-diacetyl-hexoside-*O*-glyceric acid, cyanin chloride, delphinidin 3-sophoroside-5-rhamnoside, myrtillin chloride, cyanidin 3-*O*-glucoside, cyanidin *O*-malonyl-malonylhexoside, cyanidin *O*-syringic acid, cyanidin chloride, pseudopurpurin, idaein chloride, malvidin *O*-hexoside, procyanidin B2, procyanidin B3, pelargonin chloride, procyanidin A2, keracyanin chloride, cyanidin *O*-rutinoside, cyanidin 3-*O*-glucosyl-malonylglucoside, malvidin 3-galactoside, ferulylpelargonidin di-*O*-hexosyl-*O*-pentoside chloride, peonidin, chloride, procyanidin A1, procyanidin A3, pelargonidin *O*-acetylhexoside, pelargonidin 3-*O*-malonylhexoside, cyanidin *O*-acetylhexoside, cyanidin 3-*O*-malonylhexoside, and peonidin *O*-hexoside (**Figure 9A**). Correlations between the differentially expressed MYBs, bHLHs, and differentially accumulated metabolites showed that 10 MYB and 10 bHLH TFs exhibited a significant positive correlation with 36 and 30 anthocyanins, respectively (*r* > 0.8) (**Figures 9B, C**; **Supplementary Tables 13**, **14**). As crucial regulators of plant anthocyanin biosynthesis, the two R2R3-MYB TFs of the SG5 subgroup (Podel.06G234300 and Podel.04G021100), based on phylogenetic analysis and gene function annotation, were significantly positively correlated with six anthocyanins (malvidin 3-galactoside chloride, peonidin *O*-hexoside, pelargonidin *O*-acetylhexoside, pelargonidin 3*-O*-malonylhexoside, cyanidin 3-*O*-malonylhexoside, and cyanidin chloride) and 16 anthocyanins (procyanidin A1, procyanidin A2, procyanidin A3, procyanidin B2, procyanidin B3, cyanin chloride, cyanidin *O*-rutinoside, cyanidin *O*-acetylhexoside, cyanidin 3-*O*-glucoside, cyanidin *O*-malonyl-malonylhexoside, keracyanin chloride, ferulylpelargonidin di-*O*-hexosyl-*O*-pentoside, pelargonidin 3-*O*-malonylhexoside, malvidin 3-galactoside chloride, peonidin chloride, and pseudopurpurin), respectively. The strong correlation of Podel.04G021100 with procyanidin and cyanidin indicated that one MYB (Podel.04G021100) may be involved in regulating anthocyanin biosynthesis.

## Discussion

The different colors of plant organs depend on the relative content and distribution of the three types of pigments (chlorophyll, carotenoids, and anthocyanins) (Baek et al., 2012; Sousa, 2022). A previous study reported that a high ratio of anthocyanins relative to the other two pigments (chlorophyll and carotenoids) was detected in a colored-leaf cultivar of *P. deltoides* (‘Caihong’, CHP) (Zhuang et al., 2019). Tian et al. (2021b) proposed a pigmentation mechanism in JHY by which carotenoids and anthocyanins overlay each other and are combined with a reduction in chlorophyll content, thus causing the golden color of the leaves (Tian et al., 2021a). However, the anthocyanin components and the molecular mechanisms of anthocyanin biosynthesis in colored-leaf cultivars of *P. deltoides* QHP and ZSY remained unclear (**Figures 1A, B**). The present results showed that the contents of chlorophyll and carotenoid in P and F_P were significantly lower than those in G and F_G (**Figures 1C, D** and **Supplementary Table 2**). The anthocyanin content in purple leaves of QHP and ZSY (P and F_P) was significantly higher than that in green leaves of L2025 and ZSY (G and F_G) (**Figure 1E** and **Supplementary Table 2**). These results suggested that anthocyanins were the main contributor to the purple leaf phenotype, which was consistent with previous findings (Zhang et al., 2016). In the present study, integrated metabolite and transcriptome profiling revealed the anthocyanin composition and identified DEGs involved in anthocyanin biosynthesis in purple leaves of QHP and ZSY.

Thirty-nine anthocyanin compounds were identified in the leaves of QHP and ZSY based on metabolite profile analysis (**Table 1** and **Figure 2**). Over 61.5% of these compounds were cyanidins, pelargonidins, and delphindins, which was consistent with previous findings by Ni et al. (2021). Moreover, the contents of most differential anthocyanin metabolites were upregulated in both F_P vs. F_G and P vs G (**Figures 2B, C**), suggesting that the accumulation of anthocyanins systematically provided a strong basis for the development of purple leaves in colored-leaf cultivars of *P. deltoides*, which was consistent with a previous study (Tian et al., 2021b). Tian et al. (2021b) detected 13 anthocyanin metabolites in ‘golden leaf mutant poplar variety’ (JHY) and QHP, and phenylpropanoid biosynthesis-related genes and three MYB TFs involved in anthocyanin biosynthesis were also identified. The structural genes and TFs involved in anthocyanin biosynthesis have been identified in genetic analyses of model plants, such as apple, grape, and peach (Cheng et al., 2014; Xu et al., 2017; Zhang et al., 2017; Honda and Moriya, 2018). To date, the molecular mechanism of anthocyanin accumulation in colored-leaf poplar cultivars (QHP and ZSY) has not been studied in detail. In the present study, 15 and 16 DEGs encoding key enzymes for anthocyanin biosynthesis were upregulated in F_P *vs*. F_G and P vs. G, respectively (**Figure 6** and **Supplementary Table 9**), which was consistent with the results of a previous study (Chen et al., 2021). In a previous study, 15 and 11 DEGs involved in anthocyanin biosynthesis were screened in QHP and ‘Xuanhong’, respectively, and three TFs (HY5, HYH, and TTG2) may be directly associated with anthocyanin biosynthesis in both red-leaved poplars based on the results of a combined transcriptome and proteome analysis (Chen et al., 2021). The enzyme UFGT is essential for maintaining proper production quantity, acylation, and glucosylation of anthocyanin (Muhammad et al., 2022). In the present study, three *UFGT* genes were significantly upregulated in both F_P vs. F_G and P vs. G, which was consistent with the results of the metabolite profile analysis and the expression pattern of most DEGs (**Figure 6** and **Supplementary Table 9**), indicating that these *UFGT* genes might be involved in catalyzing the formation of glycosylated anthocyanins.

Anthocyanin biosynthesis is regulated by several TFs belonging to different families, such as MYB, bHLH, WD40, AP2/ERF, bZIP, WRKY, and Dof (Rameneni et al., 2020; Yuan et al., 2020). In the current study, 5 MYB, 3 bHLH, 17 AP2/ERP, and 1 WD4 TFs showed the same expression pattern as most structural genes involved in anthocyanin biosynthesis, which were upregulated in both F_P vs. F_G and P vs G (**Figure 7** and **Supplementary Table 10**). This result demonstrated that these TFs play positive regulatory roles in anthocyanin biosynthesis. In contrast, 33 MYB, 21 bHLH, 17 AP2/ERP, 6 bZIP, and 7 WRKY exhibited the opposite expression patterns to most structural genes involved in anthocyanin biosynthesis, which were downregulated in both F_P vs F_G and P vs G (**Figure 7** and **Supplementary Table 10**). This result demonstrated that these TFs play negative regulatory roles in anthocyanin biosynthesis. Phylogenetic analysis showed that two MYB TFs, Podel.06G234300 and Podel.04G021100, belonged to the SG5 subgroup of R2R3-MYB TFs (**Figure S1**). Members of the SG5 subgroup in *Arabidopsis* are involved in regulating anthocyanin and proanthocyanidin biosynthesis (Schwinn et al., 2016).

A correlation of RNA-seq analysis and metabolite profiling confirmed that the expression patterns of certain structural genes and TFs were significantly correlated with the production of some anthocyanins (**Figure 9** and **Supplementary Tables 12**–**14**), suggesting that these genes were the primary contributors to anthocyanin accumulation in purple leaves of QHP and ZSY. In particular, one R2R3-MYB TF (Podel.04G021100) belonging to the SG5 subgroup was significantly positively correlated with 16 anthocyanins. Moreover, Podel.04G021100 showed a similar correlation pattern with three *CHS*, one *F3H*, one *F3’H*, one *DFR*, one *ANS*, and three *UFGT* genes, which were significantly upregulated in purple leaves of QHP and ZSY (P and F_P). Based on the phylogenetic analysis, the ortholog of Podel.04G021100 was *Arabidopsis* AtMYB123 (TT2) (**Figure S1**). *Arabidopsis* AtMYB123 (TT2) primarily regulates the expression of DFR and ANR to catalyze the formation of anthocyanins and proanthocyanidins (Li et al., 2019; Zhao et al., 2019). Overall, Podel.04G021100 was a key regulator of anthocyanin and proanthocyanidin biosynthesis in purple leaves of QHP and ZSY.

## Conclusion

In this study, the constituents and kinetic pattern of anthocyanin accumulation and differentially expressed anthocyanins were detected in the leaves of QHP, ZSY, and L2025 plants. Some differentially expressed structural genes and TFs associated with anthocyanin biosynthesis were identified and verified by qRT-PCR (TDKodama et al., 2018; Xi et al., 2019; Li et al., 2021; Li et al., 2022). Genes regulating the differential accumulation of anthocyanins were identified, and the critical regulatory genes involved in anthocyanin biosynthesis in purple leaves of QHP and ZSY were identified based on a correlation analysis between the RNA-seq data and metabolite profiling, and phylogenetic analysis. These results enhance our understanding of the anthocyanin composition and corresponding molecular mechanisms of anthocyanin biosynthesis in purple leaves of QHP and ZSY.

## Data availability statement

The datasets presented in this study can be found in online repositories. The names of the repository/repositories and accession number(s) can be found in the article/**Supplementary Material**.

## Ethics statement

All plants materials used in this study do not include any wild species at risk of extinction. No specific permits are required for sample collection in this study. We comply with relevant institutional, national, and international guidelines and legislation for plant study.

## Author contributions

WZ and XW designed the experiment. YA, XP, YP, YL, ZW, BY, and ZZ conducted the experiment and analyzed the data. XW wrote the manuscript. WZ and YA revised the manuscript. All authors contributed to the article and approved the submitted version.
