# Supplementary material for: Transcriptome and metabolome analyses reveal molecular mechanisms of anthocyanin-related leaf color variation in poplar (Populus deltoides) cultivars
Source: Front Plant Sci. 2023 Feb 24;14:1103468. doi: 10.3389/fpls.2023.1103468 (PMC9998943; doi:10.3389/fpls.2023.1103468)
Supplement: Supplementary file 2 [file Table_1.docx]

**Supplementary Table S1 |** Primers used in this study

| Gene name | Accession | Forward primer (5’to 3’) | Reverse primer (5’ to 3’) |
| --- | --- | --- | --- |
| PdeCHS1 | Podel.14G152900 | CGTCGATTGAGGAGATCAGAAA | TCCGGAAGTAGTAGTCAGGATAG |
| PdeCHS2 | Podel.14G153100 | CCCTGACTACTACTTTCGCATC | TCCTCAGTCAAGTGCATGTATC |
| PdeCHS3 | Podel.01G055900 | TGCTGTGATCATGGCTATCG | CGCACATTCGCTTGAACTTT |
| PdeF3H2 | Podel.05G127100 | ACCTTCTTCTCGTACCCAATTC | TCTCAATGTCCTTGCTCATCTT |
| PdeF3'H1 | Podel.13G079600 | GTGACCCAAAGGCAGATGAA | CCCAGCACCGAATGGTATAAG |
| PdeDFR | Podel.01G063100 | TTGGGTCCACCTTCACTTTC | ATCGCCATCTTGCCACTATC |
| PdeANS3 | Podel.15G029700 | CTCCACCAGACCCTCTTAAATC | ACCACCCACAAGAACCTAAC |
| PdeUFGT4 | Podel.02G260900 | CGCACAAGGCCACATAAATC | GAATGACAAACCGTCAGGAAAC |
| PdeUFGT5 | Podel.07G150600 | CCAAGCTGGTAGAGGATGTTT | TTTCATCTCTTCGCCCTTCTC |
| PdeUFGT9 | Podel.07G150700 | CCTGTGTTGATCGAGGGATTAC | CCCTTCTGGGATCTTTCCTTTC |
| MYB-1 | Podel.04G021100 | ATGGCTGACACTGAACATTCT | CGTAGAGCATCTAGTGTTCCAAT |
| MYB-2 | Podel.06G234300 | CGGAGAAGGCAAATGGAGAA | CGTCTATGGCTGTTGAGATGAG |
