# Supplementary material for: Transcriptome and metabolome analyses reveal molecular mechanisms of anthocyanin-related leaf color variation in poplar (Populus deltoides) cultivars
Source: Front Plant Sci. 2023 Feb 24;14:1103468. doi: 10.3389/fpls.2023.1103468 (PMC9998943; doi:10.3389/fpls.2023.1103468)
Supplement: Supplementary file 3 [file Table_2.docx]

**Supplementary Table 2 |** The contents of pigments in leaves of colored-leaf poplar.

| Sample | Chlorophyll a（mg/g FW） | Chlorophyll b（mg/g FW） | Total Chlorophyll （mg/g FW） | Carotenoids （mg/g FW） | Anthocyanin （mg/g FW） |
| --- | --- | --- | --- | --- | --- |
| F_G | 1.14±0.00 | 0.37±0.01 | 1.51±0.01** | 0.27±0.01 | 22.41±0.05 |
| F_P | 0.40±0.00** | 0.24±0.01** | 0.64±0.00** | 0.18±0.01** | 332.34±0.01** |
| G | 1.02±0.01 | 0.40±0.01 | 1.42±0.01** | 0.24±0.01 | 24.63±0.05 |
| P | 0.60±0.00** | 0.29±0.01** | 0.89±0.00** | 0.14±0.01** | 386.51±0.01** |
