# Supplementary material for: Transcriptome and metabolome analyses reveal molecular mechanisms of anthocyanin-related leaf color variation in poplar (Populus deltoides) cultivars
Source: Front Plant Sci. 2023 Feb 24;14:1103468. doi: 10.3389/fpls.2023.1103468 (PMC9998943; doi:10.3389/fpls.2023.1103468)
Supplement: Supplementary file 4 [file Table_3.docx]

**Supplementary Table 3 |** The differential accumulation of anthocyanins metabolites in F_P *vs.* F_G and P *vs.* G.

| Metabolite types | F_G1 | F_G2 | F_G3 | F_P1 | F_P2 | F_P3 | G1 | G2 | G3 | P1 | P2 | P3 |
| --- | --- | --- | --- | --- | --- | --- | --- | --- | --- | --- | --- | --- |
| Myrtillin chloride | 0.005 | 0.0093 | 0.0107 | 0.2145 | 0.2053 | 0.1784 | 0.0308 | 0.0404 | 0.0286 | 0.9173 | 0.8606 | 0.9371 |
| Keracyanin chloride | 0.0079 | 0.0394 | 0.0252 | 0.6592 | 0.6328 | 0.6721 | 0.1592 | 0.1487 | 0.1735 | 0.9368 | 0.9651 | 0.9225 |
| Pelargonin chloride | 0.0378 | 0.0198 | 0.0321 | 0.1071 | 0.0452 | 0.0085 | 0.6813 | 0.9302 | 0.8582 | 0.4087 | 0.3564 | 0.2842 |
| Delphinidin O-malonylhexoside | 0 | 0 | 0 | 0.0645 | 0.0904 | 0.0864 | 0.8056 | 0.8828 | 0.6027 | 0.0091 | 0.0158 | 0.0245 |
| Pelargonidin 3-O-malonylhexoside | 0.0169 | 0.0193 | 0.0151 | 0.9368 | 0.8649 | 0.8656 | 0.0057 | 0.0003 | 0.0031 | 0.8429 | 0.8886 | 0.932 |
| Cyanidin 3-O-malonylhexoside | 0.0814 | 0.0715 | 0.0572 | 0.9375 | 0.9513 | 0.8645 | 0.0107 | 0.0087 | 0.0054 | 0.5875 | 0.6365 | 0.7522 |
| Malvidin 3,5-diglucoside | 0.3094 | 0.453 | 0.3414 | 0.5837 | 0.8179 | 0.8108 | 0.7842 | 0.7945 | 0.8019 | 0.1201 | 0.177 | 0.064 |
| Cyanidin 3-O-glucosyl-malonylglucoside | 0.5358 | 0.5447 | 0.5171 | 0.1769 | 0.1199 | 0.1181 | 0.918 | 0.9107 | 0.9819 | 0.1136 | 0.0931 | 0.0183 |
| Peonidin O-hexoside | 0.0012 | 0.0046 | 0.0023 | 0.9883 | 0.8592 | 0.8195 | 0.0794 | 0.0784 | 0.0794 | 0.5011 | 0.5186 | 0.5349 |
| Pelargonidin 3-O-malonyl-malonylhexoside | 0.9714 | 0.8337 | 0.6772 | 0.6837 | 0.7942 | 0.7512 | 0.2152 | 0.1043 | 0.0432 | 0.0287 | 0.0389 | 0.1013 |
| Malvidin O-hexoside | 0.5686 | 0.4609 | 0.7483 | 0.356 | 0.2283 | 0.2813 | 0.5324 | 0.5112 | 0.4609 | 0.0409 | 0.1321 | 0.4155 |
| Procyanidin A2 | 0.0367 | 0.0232 | 0.0169 | 0.3596 | 0.4977 | 0.9092 | 0.0271 | 0.0114 | 0.0068 | 0.6028 | 0.7255 | 0.7414 |
| Cyanidin O-malonyl-malonylhexoside | 0.0984 | 0.1296 | 0.1157 | 0.1631 | 0.1426 | 0.0952 | 0.0209 | 0.0353 | 0.0179 | 0.9339 | 0.9921 | 0.96 |
| Peonidin chloride | 0.126 | 0.0698 | 0.0919 | 0.9418 | 0.733 | 0.6377 | 0.0149 | 0.0063 | 0.0194 | 0.6133 | 0.5742 | 0.5568 |
| Malvidin chloride | 0.0505 | 0.0534 | 0.1645 | 0.4185 | 0.4306 | 0.2254 | 0.5149 | 0.4209 | 0.3523 | 0.924 | 0.6862 | 0.4342 |
| Cyanidin chloride | 0 | 0 | 0 | 0.0617 | 0.4085 | 0.7373 | 0 | 0 | 0 | 0 | 0 | 0 |
| Idaein chloride | 0 | 0 | 0 | 0.2035 | 0.2715 | 0.1412 | 0 | 0 | 0 | 0.6219 | 0.6538 | 0.9367 |
| Pseudopurpurin | 0.0171 | 0.0311 | 0.0041 | 0.389 | 0.333 | 0.2812 | 0.0652 | 0.042 | 0.0901 | 0.882 | 0.9103 | 0.9552 |
| Ferulylpelargonidin di-O-hexosyl-O-pentoside | 0.589 | 0.5935 | 0.5484 | 0 | 0 | 0 | 0.6735 | 0.2387 | 0.1563 | 0 | 0 | 0 |
| Procyanidin A1 | 0.0225 | 0.0247 | 0.0186 | 0.4389 | 0.5685 | 0.9075 | 0.0152 | 0.0071 | 0.0026 | 0.4638 | 0.4836 | 0.5874 |
| Petunidin 3-O-rutinoside | 0.0055 | 0.009 | 0.0147 | 0.2732 | 0.2429 | 0.2837 | 0.9278 | 0.8737 | 0.9096 | 0.3873 | 0.364 | 0.3496 |
| Cyanidin O-rutinoside | 0.0135 | 0.0146 | 0.0207 | 0.4148 | 0.52 | 0.6912 | 0.0801 | 0.1006 | 0.1154 | 0.8484 | 0.8441 | 0.9263 |
| Cyanidin O-syringic acid | 0 | 0 | 0 | 0.2015 | 0.2586 | 0.1427 | 0 | 0 | 0 | 0.6073 | 0.7105 | 0.9547 |
| Cyanidin O-acetylhexoside | 0.0254 | 0.0109 | 0.0213 | 0.941 | 0.9431 | 0.8142 | 0.03 | 0.0315 | 0.031 | 0.5651 | 0.6033 | 0.6338 |
| Cyanidin O-diacetyl-hexoside-O-glyceric acid | 0.0578 | 0.0493 | 0.0594 | 0.2577 | 0.2158 | 0.1575 | 0.0136 | 0.0267 | 0.0028 | 0.7771 | 0.8592 | 0.9324 |
| Delphinidin chloride | 0.1292 | 0.1471 | 0.0923 | 0.0325 | 0.3385 | 0.334 | 0.4833 | 0.4832 | 0.8057 | 0.4165 | 0.3764 | 0.2351 |
| Procyanidin A3 | 0.0405 | 0.0222 | 0.0223 | 0.3984 | 0.5209 | 0.8897 | 0.0195 | 0.0063 | 0.0044 | 0.3821 | 0.4615 | 0.6621 |
| Procyanidin B2 | 0.0537 | 0.0093 | 0.0063 | 0.4058 | 0.3483 | 0.6081 | 0.0382 | 0.0023 | 0.0042 | 0.6461 | 0.9131 | 0.9148 |
| Procyanidin B3 | 0.0306 | 0.0106 | 0.0035 | 0.36 | 0.4232 | 0.4146 | 0.0257 | 0.001 | 0.0027 | 0.501 | 0.615 | 0.8263 |
| Delphinidin 3-sophoroside-5-rhamnoside | 0.0088 | 0.0086 | 0.0059 | 0.4945 | 0.4388 | 0.378 | 0.0019 | 0.0006 | 0.0012 | 0.822 | 0.8809 | 0.9586 |
| Cyanidin 3-O-glucoside | 0.0095 | 0.0064 | 0.0067 | 0.3731 | 0.4316 | 0.3499 | 0.0025 | 0.0011 | 0.0003 | 0.6615 | 0.726 | 0.9651 |
| Petunidin-3-O-glucoside chloride | 0.0077 | 0.0329 | 0.0761 | 0.1589 | 0.0952 | 0.1075 | 0.9596 | 0.5779 | 0.6104 | 0.1245 | 0.1448 | 0.2636 |
| Pelargonidin chloride | 0.7679 | 0.9836 | 0.8073 | 0 | 0 | 0 | 0.5083 | 0.2899 | 0.1299 | 0.2308 | 0.2744 | 0.2819 |
| Cyanin chloride | 0.064 | 0.0598 | 0.0343 | 0.4371 | 0.4031 | 0.3752 | 0.098 | 0.1251 | 0.0709 | 0.6793 | 0.8043 | 0.88 |
| Callistephin chloride | 0.1139 | 0.1064 | 0.0421 | 0.0539 | 0.2596 | 0.7445 | 0.6759 | 0.7237 | 0.7193 | 0.4483 | 0.3534 | 0.354 |
| Pelargonidin O-acetylhexoside | 0.0093 | 0.0137 | 0.0166 | 0.8141 | 0.7916 | 0.9832 | 0.2798 | 0.1665 | 0.1762 | 0.4452 | 0.4442 | 0.5239 |
| 3,3,4,5,5,7,8-Heptahydroxyflavone | 0.7951 | 0.722 | 0.4906 | 0.3528 | 0.29 | 0.6453 | 0.3935 | 0.3256 | 0.226 | 0.3659 | 0.052 | 0.4317 |
| Gentisin | 0.4505 | 0.3532 | 0.5761 | 0.6624 | 0.5056 | 0.5669 | 0.1878 | 0.5131 | 0.5653 | 0.6094 | 0.2036 | 0.2655 |
| Malvidin 3-galactoside chloride | 0.0185 | 0.0414 | 0.0454 | 0.9521 | 0.8282 | 0.7363 | 0.2265 | 0.2949 | 0.2059 | 0.4303 | 0.464 | 0.4399 |

Note: P, represents the leaves of QHP; G, represents the leaves of L2025; F_P represents the purple leaves of ZSY; F_G represents the green leaves of ZSY.
