# Supplementary material for: Transcriptome and metabolome analyses reveal molecular mechanisms of anthocyanin-related leaf color variation in poplar (Populus deltoides) cultivars
Source: Front Plant Sci. 2023 Feb 24;14:1103468. doi: 10.3389/fpls.2023.1103468 (PMC9998943; doi:10.3389/fpls.2023.1103468)
Supplement: Supplementary file 7 [file Table_6.docx]

**Supplementary Table 6 |** Summary statistics of RNA-seq results in the leaves of colored-leaf poplar.

| Sample | Raw_reads | Clean_reads | Total_map | Unique_map | Multi_map | Clean_bases | expressed gene | Standard_deviation | Q20 | Q30 | GC_pct |
| --- | --- | --- | --- | --- | --- | --- | --- | --- | --- | --- | --- |
| F_G1 | 44723028 | 42122310 | 34725853(82.44%) | 31842948(75.6%) | 2882905(6.84%) | 6.32G | 32819 | 64.304328 | 97.44 | 93.37 | 44.49 |
| F_G2 | 46000070 | 41748254 | 37571502(90.0%) | 34716661(83.16%) | 2854841(6.84%) | 6.26G | 32704 | 63.246129 | 97.59 | 93.57 | 43.21 |
| F_G3 | 46112070 | 44189218 | 35452523(80.23%) | 32399426(73.32%) | 3053097(6.91%) | 6.63G | 34047 | 65.660723 | 97.43 | 93.51 | 45.08 |
| F_P1 | 44468864 | 43083206 | 38057811(88.34%) | 35280916(81.89%) | 2776895(6.45%) | 6.46G | 32331 | 67.499664 | 97.55 | 93.65 | 44.07 |
| F_P2 | 44862662 | 43267760 | 38156607(88.19%) | 35340434(81.68%) | 2816173(6.51%) | 6.49G | 32321 | 67.411747 | 97.43 | 93.42 | 44.03 |
| F_P3 | 46038432 | 44584822 | 39470763(88.53%) | 36557099(81.99%) | 2913664(6.54%) | 6.69G | 32337 | 67.379418 | 97.45 | 93.42 | 44.01 |
| G1 | 45061512 | 43794964 | 37737038(86.17%) | 35049537(80.03%) | 2687501(6.14%) | 6.57G | 34290 | 67.661881 | 97.23 | 92.38 | 44.47 |
| G2 | 42021546 | 40623170 | 35132698(86.48%) | 32610488(80.28%) | 2522210(6.21%) | 6.09G | 34098 | 67.977085 | 97.73 | 93.48 | 44.56 |
| G3 | 43608288 | 41598682 | 36172558(86.96%) | 33682168(80.97%) | 2490390(5.99%) | 6.24G | 33214 | 66.283656 | 97.79 | 93.7 | 43.32 |
| P1 | 44506378 | 41718522 | 38477228(92.23%) | 35680435(85.53%) | 2796793(6.7%) | 6.26G | 33014 | 66.229468 | 97.87 | 93.82 | 42.69 |
| P2 | 44845928 | 42429434 | 39335302(92.71%) | 36493249(86.01%) | 2842053(6.7%) | 6.36G | 33300 | 66.402159 | 97.82 | 93.72 | 43.31 |
| P3 | 43539058 | 41235222 | 38094766(92.38%) | 35354014(85.74%) | 2740752(6.65%) | 6.19G | 32998 | 67.236575 | 97.77 | 93.57 | 43.31 |
