# Supplementary material for: Transcriptome and metabolome analyses reveal molecular mechanisms of anthocyanin-related leaf color variation in poplar (Populus deltoides) cultivars
Source: Front Plant Sci. 2023 Feb 24;14:1103468. doi: 10.3389/fpls.2023.1103468 (PMC9998943; doi:10.3389/fpls.2023.1103468)
Supplement: Supplementary file 10 [file Table_9.docx]

**Supplementary Table 9** The expression level of differential structure genes associated with anthocyanin biosynthesis in leaves of colored-leaf poplar.

| Gene_id | ID | F_G1 | F_G2 | F_G3 | F_P1 | F_P2 | F_P3 | G1 | G2 | G3 | P1 | P2 | P3 |
| --- | --- | --- | --- | --- | --- | --- | --- | --- | --- | --- | --- | --- | --- |
| Podel.04G076900 | PdeANS1 | 5.88 | 3.41 | 6.38 | 7.52 | 8.84 | 7.80 | 11.83 | 12.14 | 9.38 | 3.23 | 2.76 | 2.34 |
| Podel.04G077100 | PdeANS2 | 1.00 | 1.05 | 1.18 | 2.10 | 2.31 | 1.30 | 3.20 | 2.63 | 3.54 | 1.16 | 0.93 | 0.77 |
| Podel.15G029700 | PdeANS3 | 5.96 | 10.30 | 9.03 | 46.04 | 44.95 | 48.10 | 9.63 | 7.54 | 9.08 | 61.84 | 53.56 | 59.46 |
| Podel.13G181200 | PdeANS4 | 1.70 | 0.99 | 2.40 | 3.99 | 3.47 | 4.31 | 7.72 | 7.82 | 7.11 | 4.92 | 6.31 | 6.01 |
| Podel.06G180700 | PdeANS5 | 10.63 | 10.56 | 11.61 | 3.76 | 3.45 | 3.36 | 13.44 | 14.83 | 11.49 | 6.04 | 7.24 | 5.33 |
| Podel.09G138600 | PdeANS6 | 6.60 | 3.19 | 3.04 | 0.19 | 0.03 | 0.00 | 5.69 | 7.28 | 9.60 | 0.64 | 0.63 | 0.74 |
| Podel.14G152900 | PdeCHS1 | 29.47 | 22.67 | 24.57 | 67.76 | 111.98 | 80.15 | 17.32 | 21.19 | 15.05 | 51.41 | 78.41 | 73.59 |
| Podel.14G153100 | PdeCHS2 | 167.48 | 183.12 | 180.96 | 1063.50 | 1058.76 | 1036.44 | 141.69 | 138.63 | 126.96 | 705.23 | 810.09 | 829.43 |
| Podel.01G055900 | PdeCHS3 | 28.99 | 26.89 | 27.56 | 51.03 | 51.89 | 44.48 | 35.15 | 28.60 | 25.43 | 70.80 | 62.23 | 73.86 |
| Podel.01G055700 | PdeCHS4 | 20.52 | 22.51 | 19.66 | 19.46 | 21.77 | 20.74 | 18.32 | 14.31 | 14.98 | 37.98 | 44.08 | 41.37 |
| Podel.03G190000 | PdeCHS5 | 0.64 | 0.46 | 0.23 | 0.04 | 0.00 | 0.00 | 3.19 | 3.26 | 3.15 | 0.12 | 0.50 | 0.08 |
| Podel.14G155700 | PdeCHS6 | 8.34 | 6.03 | 8.50 | 25.27 | 27.89 | 24.14 | 11.83 | 9.72 | 7.28 | 20.87 | 20.19 | 22.19 |
| Podel.01G063100 | PdeDFR | 16.57 | 18.02 | 13.83 | 29.91 | 30.75 | 32.72 | 13.00 | 12.53 | 15.89 | 29.22 | 24.63 | 26.83 |
| Podel.09G071100 | PdeF3'5'H1 | 1.93 | 3.47 | 2.07 | 0.03 | 0.00 | 0.03 | 8.36 | 5.90 | 8.02 | 0.19 | 0.24 | 0.32 |
| Podel.05G127000 | PdeF3'5'H2 | 5.92 | 5.31 | 5.64 | 1.35 | 0.55 | 1.26 | 2.16 | 1.10 | 0.55 | 2.63 | 5.16 | 3.16 |
| Podel.05G126800 | PdeF3H1 | 2.39 | 1.48 | 2.04 | 0.33 | 0.87 | 0.60 | 11.75 | 11.37 | 13.02 | 1.52 | 1.52 | 1.15 |
| Podel.13G079600 | PdeF3'H1 | 132.95 | 129.80 | 144.92 | 549.78 | 526.25 | 517.89 | 163.92 | 156.92 | 120.47 | 382.19 | 443.58 | 439.82 |
| Podel.05G127100 | PdeF3H2 | 121.58 | 118.20 | 119.30 | 29.40 | 33.10 | 26.28 | 76.54 | 73.55 | 71.80 | 40.59 | 46.91 | 42.84 |
| Podel.01G178900 | PdeF3'H2 | 25.77 | 24.44 | 21.85 | 5.18 | 5.85 | 4.31 | 21.52 | 21.46 | 21.92 | 7.85 | 8.69 | 8.41 |
| Podel.11G147200 | PdeF3H3 | 1.93 | 3.72 | 1.93 | 0.82 | 1.38 | 1.43 | 1.85 | 0.76 | 1.05 | 0.47 | 0.32 | 0.28 |
| Podel.06G110000 | PdeF3'H3 | 0.25 | 0.49 | 0.82 | 0.16 | 0.58 | 0.75 | 0.04 | 0.00 | 0.00 | 0.29 | 0.17 | 0.43 |
| Podel.01G469000 | PdeF3H4 | 1.09 | 1.42 | 1.75 | 0.05 | 0.10 | 0.40 | 3.23 | 3.69 | 5.71 | 0.53 | 0.56 | 0.10 |
| Podel.01G231000 | PdeFLS1 | 0.82 | 0.00 | 0.76 | 0.00 | 0.00 | 0.00 | 0.39 | 0.53 | 0.06 | 0.00 | 0.00 | 0.00 |
| Podel.04G144800 | PdeFLS2 | 18.31 | 17.31 | 16.94 | 62.01 | 65.39 | 62.90 | 109.44 | 108.03 | 96.17 | 114.28 | 117.10 | 115.98 |
| Podel.07G150400 | PdeFLS3 | 0.62 | 0.37 | 0.53 | 2.57 | 3.24 | 2.63 | 0.13 | 0.00 | 0.00 | 1.93 | 1.65 | 1.36 |
| Podel.07G151300 | PdeFLS4 | 30.69 | 34.99 | 31.77 | 68.15 | 66.79 | 67.78 | 5.63 | 6.46 | 6.57 | 17.83 | 14.28 | 16.48 |
| Podel.14G154000 | PdeUFGT1 | 3.16 | 3.44 | 4.76 | 4.23 | 4.51 | 5.34 | 7.71 | 6.54 | 5.60 | 2.80 | 2.07 | 2.91 |
| Podel.09G098100 | PdeUFGT10 | 29.79 | 34.91 | 31.08 | 7.07 | 7.54 | 7.74 | 39.65 | 39.78 | 33.43 | 15.10 | 16.56 | 17.43 |
| Podel.07G152400 | PdeUFGT11 | 4.03 | 2.61 | 8.75 | 9.12 | 11.10 | 14.78 | 0.67 | 1.81 | 0.16 | 0.57 | 1.76 | 1.38 |
| Podel.16G056400 | PdeUFGT2 | 0.00 | 0.00 | 0.04 | 0.31 | 0.00 | 0.04 | 2.63 | 3.94 | 3.87 | 0.07 | 0.17 | 0.21 |
| Podel.06G057500 | PdeUFGT3 | 0.51 | 1.48 | 1.87 | 9.38 | 12.13 | 11.19 | 0.08 | 0.17 | 0.00 | 0.41 | 0.33 | 0.76 |
| Podel.02G260900 | PdeUFGT4 | 24.80 | 25.71 | 29.19 | 64.45 | 63.97 | 68.36 | 11.38 | 10.60 | 7.11 | 28.12 | 31.39 | 27.76 |
| Podel.07G150600 | PdeUFGT5 | 69.16 | 80.92 | 76.82 | 182.74 | 192.84 | 186.71 | 47.03 | 48.73 | 37.87 | 129.97 | 138.47 | 124.73 |
| Podel.09G097700 | PdeUFGT6 | 9.79 | 8.42 | 9.44 | 1.45 | 1.91 | 1.25 | 31.89 | 34.43 | 33.59 | 5.23 | 5.71 | 6.52 |
| Podel.T002500 | PdeUFGT7 | 0.00 | 0.00 | 0.00 | 0.16 | 0.16 | 0.31 | 0.00 | 0.00 | 0.00 | 0.00 | 0.00 | 0.00 |
| Podel.09G098000 | PdeUFGT8 | 1.58 | 2.58 | 3.01 | 0.69 | 0.72 | 0.36 | 6.46 | 6.51 | 3.67 | 1.41 | 1.25 | 1.11 |
| Podel.07G150700 | PdeUFGT9 | 81.00 | 79.60 | 92.14 | 164.76 | 158.12 | 157.70 | 41.80 | 39.14 | 34.88 | 117.02 | 128.62 | 126.70 |
