# Supplementary material for: Transcriptome and metabolome analyses reveal molecular mechanisms of anthocyanin-related leaf color variation in poplar (Populus deltoides) cultivars
Source: Front Plant Sci. 2023 Feb 24;14:1103468. doi: 10.3389/fpls.2023.1103468 (PMC9998943; doi:10.3389/fpls.2023.1103468)
Supplement: Supplementary file 11 [file Table_10.docx]

**Supplementary Table 10 |** The number of TFs with differential expression involved in regulating anthocyanin biosynthesis in leaves of colored-leaf poplar.

| Family | F_G VS F_P | | G VS P | |
| --- | --- | --- | --- | --- |
|  | Down | UP | Down | UP |
| MYB | 64 | 11 | 57 | 19 |
| bHLH | 34 | 5 | 24 | 13 |
| WRKY | 20 | 6 | 24 | 6 |
| AP2 | 46 | 6 | 32 | 6 |
| GRAS | 14 | 2 | 16 | 8 |
| Dof | 21 | 1 | 20 | 0 |
| Aux/IAA | 21 | 1 | 21 | 5 |
| SBP | 4 | 2 | 6 | 2 |
| Arf | 2 | 0 | 7 | 0 |
| HD-ZIP | 3 | 0 | 2 | 0 |
| LIM | 4 | 1 | 5 | 4 |
| B3 | 13 | 1 | 8 | 10 |
| TCP | 5 | 1 | 8 | 3 |
| Homeobox | 12 | 2 | 8 | 7 |
| WD40 | 1 | 0 | 6 | 6 |
| ZF-HD | 5 | 1 | 2 | 1 |
| PHD | 1 | 0 | 4 | 2 |
| bZIP | 9 | 11 | 11 | 12 |
