# Supplementary material for: Transcriptome and metabolome analyses reveal molecular mechanisms of anthocyanin-related leaf color variation in poplar (Populus deltoides) cultivars
Source: Front Plant Sci. 2023 Feb 24;14:1103468. doi: 10.3389/fpls.2023.1103468 (PMC9998943; doi:10.3389/fpls.2023.1103468)
Supplement: Supplementary file 12 [file Table_11.docx]

**Supplementary Table 11** | Determination of the relative expression of structural genes and two MYB transcription factors involved in the anthocyanin biosynthesis pathway by qRT PCR.

| Gene | ID | F_G | Standard deviation | F_P | Standard deviation | G | Standard deviation | P | Standard deviation |
| --- | --- | --- | --- | --- | --- | --- | --- | --- | --- |
| Podel.14G152900 | PdeCHS1 | 1 | 0.0743 | 3.7167 | 0.1579 | 1 | 0.1650 | 4.2221 | 0.8849 |
| Podel.14G153100 | PdeCHS2 | 1 | 0.0396 | 1.4075 | 0.2859 | 1 | 0.0454 | 1.6921 | 0.0344 |
| Podel.01G055900 | PdeCHS3 | 1 | 0.0317 | 1.2232 | 0.0057 | 1 | 0.1895 | 1.8049 | 0.0409 |
| Podel.05G127100 | PdeF3H2 | 1 | 0.0282 | 3.9347 | 0.0592 | 1 | 0.0936 | 2.8062 | 0.4982 |
| Podel.13G079600 | PdeF3'H1 | 1 | 0.0357 | 2.6814 | 0.0761 | 1 | 0.0529 | 3.0604 | 0.7738 |
| Podel.01G063100 | PdeDFR | 1 | 0.0086 | 4.3679 | 0.0929 | 1 | 0.0835 | 2.5814 | 0.3102 |
| Podel.15G029700 | PdeANS3 | 1 | 0.0205 | 2.9489 | 0.0761 | 1 | 0.1013 | 1.715 | 0.1534 |
| Podel.02G260900 | PdeUFGT4 | 1 | 0.0444 | 3.0927 | 0.0616 | 1 | 0.1789 | 1.6768 | 0.24 |
| Podel.07G150600 | PdeUFGT5 | 1 | 0.0142 | 29.9308 | 0.6775 | 1 | 0.0131 | 9.2138 | 0.6752 |
| Podel.07G150700 | PdeUFGT9 | 1 | 0.0172 | 5.8911 | 0.3162 | 1 | 0.1694 | 3.4794 | 0.5785 |
| Podel.04G021100 | PdeMYB1 | 1 | 0.0256 | 5.8911 | 0.3162 | 1 | 0.1122 | 2.7597 | 0.9467 |
| Podel.06G234300 | PdeMYB2 | 1 | 0.0113 | 0.0502 | 0.0011 | 1 | 0.0566 | 0.2286 | 0.0224 |
