# Supplementary material for: Transcriptome and metabolome analyses reveal molecular mechanisms of anthocyanin-related leaf color variation in poplar (Populus deltoides) cultivars
Source: Front Plant Sci. 2023 Feb 24;14:1103468. doi: 10.3389/fpls.2023.1103468 (PMC9998943; doi:10.3389/fpls.2023.1103468)
Supplement: Supplementary file 13 [file Table_12.docx]

**Supplementary Table 12 |** Connection network between the structure genes and anthocyanin metabolites.

| Var1 | Var2 | cor | p_value |
| --- | --- | --- | --- |
| PdeANS3 | Cyanidin O-diacetyl-hexoside-O-glyceric acid | 0.837703107 | 0.000670752 |
| PdeCHS3 | Cyanidin O-diacetyl-hexoside-O-glyceric acid | 0.930681787 | 1.12109E-05 |
| PdeANS3 | Delphinidin 3-sophoroside-5-rhamnoside | 0.954635208 | 1.40192E-06 |
| PdeCHS3 | Delphinidin 3-sophoroside-5-rhamnoside | 0.98008777 | 2.38442E-08 |
| PdeANS3 | Cyanin chloride | 0.936118434 | 7.52175E-06 |
| PdeCHS3 | Cyanin chloride | 0.97204543 | 1.28284E-07 |
| PdeANS3 | Cyanidin 3-O-glucoside | 0.940055618 | 5.50937E-06 |
| PdeCHS3 | Cyanidin 3-O-glucoside | 0.975131447 | 7.18484E-08 |
| PdeCHS3 | Cyanidin O-malonyl-malonylhexoside | 0.870142887 | 0.000232912 |
| PdeANS3 | Cyanidin O-syringic acid | 0.846399839 | 0.000517206 |
| PdeCHS3 | Cyanidin O-syringic acid | 0.939596377 | 5.71918E-06 |
| PdeANS3 | Keracyanin chloride | 0.98295333 | 1.10172E-08 |
| PdeCHS2 | Keracyanin chloride | 0.848666386 | 0.000482084 |
| PdeCHS3 | Keracyanin chloride | 0.954921451 | 1.3589E-06 |
| PdeDFR | Keracyanin chloride | 0.834521425 | 0.000734962 |
| PdeF3'H1 | Keracyanin chloride | 0.86371615 | 0.000293216 |
| PdeANS3 | Cyanidin O-rutinoside | 0.970527107 | 1.6669E-07 |
| PdeCHS2 | Cyanidin O-rutinoside | 0.808177618 | 0.001467594 |
| PdeCHS3 | Cyanidin O-rutinoside | 0.94758935 | 2.85121E-06 |
| PdeDFR | Cyanidin O-rutinoside | 0.813882102 | 0.001274959 |
| PdeF3'H1 | Cyanidin O-rutinoside | 0.817068292 | 0.001176194 |
| PdeF3H2 | Ferulylpelargonidin di-O-hexosyl-O-pentoside | 0.895205278 | 8.32808E-05 |
| PdeCHS1 | Malvidin 3-galactoside chloride | 0.822576826 | 0.001019445 |
| PdeCHS2 | Malvidin 3-galactoside chloride | 0.915510413 | 2.93838E-05 |
| PdeDFR | Malvidin 3-galactoside chloride | 0.840444063 | 0.000618996 |
| PdeF3'H1 | Malvidin 3-galactoside chloride | 0.928346508 | 1.31781E-05 |
| PdeUFGT4 | Malvidin 3-galactoside chloride | 0.815764552 | 0.001215867 |
| PdeUFGT5 | Malvidin 3-galactoside chloride | 0.877921377 | 0.00017337 |
| PdeANS3 | Cyanidin O-acetylhexoside | 0.878889972 | 0.000166883 |
| PdeCHS1 | Cyanidin O-acetylhexoside | 0.934091695 | 8.76269E-06 |
| PdeCHS2 | Cyanidin O-acetylhexoside | 0.995275057 | 1.83997E-11 |
| PdeDFR | Cyanidin O-acetylhexoside | 0.953253736 | 1.62497E-06 |
| PdeF3'H1 | Cyanidin O-acetylhexoside | 0.993533493 | 8.80872E-11 |
| PdeUFGT4 | Cyanidin O-acetylhexoside | 0.851174426 | 0.000445408 |
| PdeUFGT5 | Cyanidin O-acetylhexoside | 0.964276074 | 4.3153E-07 |
| PdeUFGT9 | Cyanidin O-acetylhexoside | 0.925716735 | 1.57096E-05 |
| PdeANS3 | Cyanidin 3-O-malonylhexoside | 0.896213702 | 7.94892E-05 |
| PdeCHS1 | Cyanidin 3-O-malonylhexoside | 0.942514114 | 4.4873E-06 |
| PdeCHS2 | Cyanidin 3-O-malonylhexoside | 0.997654686 | 5.56618E-13 |
| PdeDFR | Cyanidin 3-O-malonylhexoside | 0.963134097 | 5.04082E-07 |
| PdeF3'H1 | Cyanidin 3-O-malonylhexoside | 0.991863755 | 2.76995E-10 |
| PdeUFGT4 | Cyanidin 3-O-malonylhexoside | 0.849744801 | 0.000466038 |
| PdeUFGT5 | Cyanidin 3-O-malonylhexoside | 0.967984203 | 2.51048E-07 |
| PdeUFGT9 | Cyanidin 3-O-malonylhexoside | 0.944840759 | 3.66431E-06 |
| PdeANS3 | Myrtillin chloride | 0.842872941 | 0.000575768 |
| PdeCHS3 | Myrtillin chloride | 0.934646084 | 8.40827E-06 |
| PdeANS3 | Idaein chloride | 0.855299137 | 0.00038984 |
| PdeCHS3 | Idaein chloride | 0.948415826 | 2.63708E-06 |
| Pseudopurpurin | PdeANS3 | 0.90279588 | 5.79408E-05 |
| Procyanidin B2 | PdeANS3 | 0.937520536 | 6.74788E-06 |
| Procyanidin B3 | PdeANS3 | 0.952309677 | 1.79294E-06 |
| Procyanidin A2 | PdeANS3 | 0.928845515 | 1.27365E-05 |
| Peonidin chloride | PdeANS3 | 0.890281007 | 0.000103882 |
| Procyanidin A1 | PdeANS3 | 0.88806336 | 0.000114372 |
| Procyanidin A3 | PdeANS3 | 0.869196334 | 0.000241125 |
| Pelargonidin 3-O-malonylhexoside | PdeANS3 | 0.974579678 | 8.01062E-08 |
| Peonidin O-hexoside | PdeANS3 | 0.83175597 | 0.000794544 |
| Procyanidin B3 | PdeCHS1 | 0.801543921 | 0.001718893 |
| Procyanidin A2 | PdeCHS1 | 0.830092842 | 0.000832132 |
| Peonidin chloride | PdeCHS1 | 0.879818049 | 0.000160846 |
| Procyanidin A1 | PdeCHS1 | 0.881552905 | 0.000150021 |
| Procyanidin A3 | PdeCHS1 | 0.866893576 | 0.00026205 |
| Pelargonidin O-acetylhexoside | PdeCHS1 | 0.840389433 | 0.000619996 |
| Pelargonidin 3-O-malonylhexoside | PdeCHS1 | 0.899271333 | 6.88138E-05 |
| Peonidin O-hexoside | PdeCHS1 | 0.890467418 | 0.000103036 |
| Procyanidin B3 | PdeCHS2 | 0.822765687 | 0.001014373 |
| Procyanidin A2 | PdeCHS2 | 0.870520174 | 0.0002297 |
| Peonidin chloride | PdeCHS2 | 0.974564295 | 8.03468E-08 |
| Procyanidin A1 | PdeCHS2 | 0.933898952 | 8.88864E-06 |
| Procyanidin A3 | PdeCHS2 | 0.910873033 | 3.80774E-05 |
| Pelargonidin O-acetylhexoside | PdeCHS2 | 0.930908684 | 1.10329E-05 |
| Pelargonidin 3-O-malonylhexoside | PdeCHS2 | 0.968567946 | 2.29206E-07 |
| Peonidin O-hexoside | PdeCHS2 | 0.980026511 | 2.42107E-08 |
| Pseudopurpurin | PdeCHS3 | 0.964172092 | 4.3777E-07 |
| Procyanidin B2 | PdeCHS3 | 0.926899271 | 1.45277E-05 |
| Procyanidin B3 | PdeCHS3 | 0.956334177 | 1.16168E-06 |
| Procyanidin A2 | PdeCHS3 | 0.831931666 | 0.000790651 |
| Pelargonidin 3-O-malonylhexoside | PdeCHS3 | 0.886884733 | 0.000120274 |
| Procyanidin A2 | PdeDFR | 0.876354145 | 0.000184282 |
| Peonidin chloride | PdeDFR | 0.945143827 | 3.56658E-06 |
| Procyanidin A1 | PdeDFR | 0.936901 | 7.08164E-06 |
| Procyanidin A3 | PdeDFR | 0.90602072 | 4.92202E-05 |
| Pelargonidin O-acetylhexoside | PdeDFR | 0.888046943 | 0.000114452 |
| Pelargonidin 3-O-malonylhexoside | PdeDFR | 0.943952388 | 3.96304E-06 |
| Peonidin O-hexoside | PdeDFR | 0.932559188 | 9.80421E-06 |
| Procyanidin B3 | PdeF3'H1 | 0.829690807 | 0.000841421 |
| Procyanidin A2 | PdeF3'H1 | 0.868891817 | 0.000243815 |
| Peonidin chloride | PdeF3'H1 | 0.971947961 | 1.30515E-07 |
| Procyanidin A1 | PdeF3'H1 | 0.925578041 | 1.5853E-05 |
| Procyanidin A3 | PdeF3'H1 | 0.902659927 | 5.83333E-05 |
| Pelargonidin O-acetylhexoside | PdeF3'H1 | 0.937247976 | 6.89314E-06 |
| Pelargonidin 3-O-malonylhexoside | PdeF3'H1 | 0.97110365 | 1.51159E-07 |
| Peonidin O-hexoside | PdeF3'H1 | 0.981842975 | 1.5076E-08 |
| Peonidin chloride | PdeUFGT4 | 0.828928149 | 0.000859262 |
| Pelargonidin O-acetylhexoside | PdeUFGT4 | 0.831280199 | 0.00080516 |
| Peonidin O-hexoside | PdeUFGT4 | 0.863531857 | 0.000295108 |
| Procyanidin A2 | PdeUFGT5 | 0.803184663 | 0.001653883 |
| Peonidin chloride | PdeUFGT5 | 0.949958268 | 2.27165E-06 |
| Procyanidin A1 | PdeUFGT5 | 0.896660432 | 7.78534E-05 |
| Procyanidin A3 | PdeUFGT5 | 0.86279094 | 0.000302812 |
| Pelargonidin O-acetylhexoside | PdeUFGT5 | 0.891557446 | 9.81963E-05 |
| Pelargonidin 3-O-malonylhexoside | PdeUFGT5 | 0.904368336 | 5.35489E-05 |
| Peonidin O-hexoside | PdeUFGT5 | 0.94986448 | 2.29265E-06 |
| Peonidin chloride | PdeUFGT9 | 0.93964506 | 5.69665E-06 |
| Procyanidin A1 | PdeUFGT9 | 0.86458055 | 0.000284466 |
| Procyanidin A3 | PdeUFGT9 | 0.84184967 | 0.000593685 |
| Pelargonidin O-acetylhexoside | PdeUFGT9 | 0.810610423 | 0.001382889 |
| Pelargonidin 3-O-malonylhexoside | PdeUFGT9 | 0.892806556 | 9.2871E-05 |
| Peonidin O-hexoside | PdeUFGT9 | 0.897999248 | 7.31088E-05 |
| PdeANS3 | Malvidin O-hexoside | -0.817731382 | 0.001156403 |
| PdeF3H2 | Keracyanin chloride | -0.851681685 | 0.000438261 |
| PdeF3H2 | Cyanidin O-rutinoside | -0.800491322 | 0.00176162 |
| PdeANS3 | Cyanidin 3-O-glucosyl-malonylglucoside | -0.89453776 | 8.58677E-05 |
| PdeCHS1 | Cyanidin 3-O-glucosyl-malonylglucoside | -0.854580661 | 0.000399109 |
| PdeCHS2 | Cyanidin 3-O-glucosyl-malonylglucoside | -0.877217499 | 0.000178207 |
| PdeCHS3 | Cyanidin 3-O-glucosyl-malonylglucoside | -0.818774922 | 0.001125776 |
| PdeDFR | Cyanidin 3-O-glucosyl-malonylglucoside | -0.885476106 | 0.000127638 |
| PdeF3'H1 | Cyanidin 3-O-glucosyl-malonylglucoside | -0.856725408 | 0.000371937 |
| PdeUFGT5 | Cyanidin 3-O-glucosyl-malonylglucoside | -0.879297158 | 0.000164213 |
| PdeUFGT9 | Cyanidin 3-O-glucosyl-malonylglucoside | -0.933114662 | 9.41599E-06 |
| PdeANS3 | Ferulylpelargonidin di-O-hexosyl-O-pentoside | -0.837996143 | 0.000665064 |
| PdeCHS2 | Ferulylpelargonidin di-O-hexosyl-O-pentoside | -0.818470935 | 0.001134632 |
| PdeDFR | Ferulylpelargonidin di-O-hexosyl-O-pentoside | -0.801797423 | 0.001708723 |
| PdeF3'H1 | Ferulylpelargonidin di-O-hexosyl-O-pentoside | -0.823816456 | 0.000986507 |
| PdeF3H2 | Malvidin 3-galactoside chloride | -0.904443313 | 5.33462E-05 |
| PdeF3H2 | Cyanidin O-acetylhexoside | -0.873566978 | 0.000205008 |
| PdeF3H2 | Cyanidin 3-O-malonylhexoside | -0.844515481 | 0.000547889 |
| Peonidin chloride | PdeF3H2 | -0.815477955 | 0.001224725 |
| Procyanidin A1 | PdeF3H2 | -0.830445595 | 0.000824047 |
| Procyanidin A3 | PdeF3H2 | -0.803059724 | 0.001658766 |
| Pelargonidin O-acetylhexoside | PdeF3H2 | -0.913753707 | 3.24698E-05 |
| Pelargonidin 3-O-malonylhexoside | PdeF3H2 | -0.863475443 | 0.000295689 |
| Peonidin O-hexoside | PdeF3H2 | -0.888717208 | 0.000111197 |
