# Supplementary material for: Transcriptome and metabolome analyses reveal molecular mechanisms of anthocyanin-related leaf color variation in poplar (Populus deltoides) cultivars
Source: Front Plant Sci. 2023 Feb 24;14:1103468. doi: 10.3389/fpls.2023.1103468 (PMC9998943; doi:10.3389/fpls.2023.1103468)
Supplement: Supplementary file 14 [file Table_13.docx]

**Supplementary Table 13 |** Connection network between MYB TFs and anthocyanin metabolites.

| Var1 | Var2 | Cor | p_value |
| --- | --- | --- | --- |
| Podel.08G221200 | Cyanidin 3-O-malonylhexoside | 0.951686233 | 1.91121E-06 |
| Podel.14G127200 | Cyanidin 3-O-malonylhexoside | 0.984317124 | 7.27769E-09 |
| Podel.08G221200 | Cyanidin O-acetylhexoside | 0.948700606 | 2.56633E-06 |
| Podel.14G127200 | Cyanidin O-acetylhexoside | 0.994550444 | 3.75066E-11 |
| Podel.08G221200 | Pelargonidin 3-O-malonylhexoside | 0.861366899 | 0.000318058 |
| Podel.14G127200 | Pelargonidin 3-O-malonylhexoside | 0.963825226 | 4.59107E-07 |
| Podel.08G221200 | Pelargonidin O-acetylhexoside | 0.89779352 | 7.38227E-05 |
| Podel.14G127200 | Pelargonidin O-acetylhexoside | 0.944410259 | 3.80676E-06 |
| Podel.08G221200 | Peonidin chloride | 0.926391222 | 1.50266E-05 |
| Podel.14G127200 | Peonidin chloride | 0.96801579 | 2.49825E-07 |
| Podel.06G106200 | Cyanidin 3-O-glucosyl-malonylglucoside | 0.973556426 | 9.74123E-08 |
| Podel.17G037900 | Cyanidin 3-O-glucosyl-malonylglucoside | 0.945259902 | 3.5297E-06 |
| Podel.06G163300 | Cyanidin 3-O-glucosyl-malonylglucoside | 0.93606072 | 7.55504E-06 |
| Podel.07G028800 | Cyanidin 3-O-glucosyl-malonylglucoside | 0.86618452 | 0.00026877 |
| Podel.09G121500 | Cyanidin 3-O-glucosyl-malonylglucoside | 0.922478477 | 1.93378E-05 |
| Podel.02G205100 | Cyanidin 3-O-glucosyl-malonylglucoside | 0.951118634 | 2.0242E-06 |
| Podel.14G127200 | Cyanidin O-rutinoside | 0.812163353 | 0.001330818 |
| Podel.06G163300 | Petunidin 3-O-rutinoside | 0.801535657 | 0.001719226 |
| Podel.17G037900 | Delphinidin O-malonylhexoside | 0.906908878 | 4.701E-05 |
| Podel.06G163300 | Delphinidin O-malonylhexoside | 0.899363971 | 6.8509E-05 |
| Podel.07G028800 | Delphinidin O-malonylhexoside | 0.881306311 | 0.000151524 |
| Podel.17G037900 | Pelargonin chloride | 0.818643394 | 0.001129601 |
| Podel.06G163300 | Pelargonin chloride | 0.857671454 | 0.000360418 |
| Podel.07G028800 | Pelargonin chloride | 0.838329609 | 0.000658637 |
| Podel.17G037900 | Petunidin-3-O-glucoside chloride | 0.850131712 | 0.000460383 |
| Podel.06G163300 | Petunidin-3-O-glucoside chloride | 0.838404802 | 0.000657195 |
| Podel.07G028800 | Petunidin-3-O-glucoside chloride | 0.834867371 | 0.000727759 |
| Podel.02G040700 | Pelargonidin 3-O-malonyl-malonylhexoside | 0.815980262 | 0.001209233 |
| Podel.08G221200 | Peonidin O-hexoside | 0.945255149 | 3.53121E-06 |
| Podel.14G127200 | Peonidin O-hexoside | 0.987228243 | 2.61961E-09 |
| Podel.14G127200 | Keracyanin chloride | 0.866263716 | 0.000268012 |
| Podel.08G221200 | Malvidin 3-galactoside chloride | 0.881688488 | 0.0001492 |
| Podel.14G127200 | Malvidin 3-galactoside chloride | 0.947830338 | 2.7874E-06 |
| Procyanidin A3 | Podel.04G021100 | 0.816142607 | 0.001204259 |
| Procyanidin A1 | Podel.04G021100 | 0.847762014 | 0.000495869 |
| Procyanidin A3 | Podel.08G221200 | 0.851370603 | 0.000442634 |
| Procyanidin A1 | Podel.08G221200 | 0.878848136 | 0.000167159 |
| Procyanidin A3 | Podel.14G127200 | 0.878568079 | 0.000169017 |
| Procyanidin A1 | Podel.14G127200 | 0.909281776 | 4.1486E-05 |
| Procyanidin B3 | Podel.14G127200 | 0.810094583 | 0.001400527 |
| Procyanidin A2 | Podel.14G127200 | 0.846371489 | 0.000517657 |
| Podel.06G106200 | Cyanidin 3-O-malonylhexoside | -0.89569819 | 8.14102E-05 |
| Podel.09G121500 | Cyanidin 3-O-malonylhexoside | -0.971528886 | 1.4046E-07 |
| Podel.02G205100 | Cyanidin 3-O-malonylhexoside | -0.93760042 | 6.70576E-06 |
| Podel.06G106200 | Cyanidin O-acetylhexoside | -0.865691184 | 0.000273524 |
| Podel.09G121500 | Cyanidin O-acetylhexoside | -0.960482338 | 7.10208E-07 |
| Podel.02G205100 | Cyanidin O-acetylhexoside | -0.916253835 | 2.81496E-05 |
| Podel.06G106200 | Pelargonidin 3-O-malonylhexoside | -0.890649632 | 0.000102214 |
| Podel.09G121500 | Pelargonidin 3-O-malonylhexoside | -0.981696307 | 1.56909E-08 |
| Podel.02G205100 | Pelargonidin 3-O-malonylhexoside | -0.913231137 | 3.34354E-05 |
| Podel.09G121500 | Pelargonidin O-acetylhexoside | -0.870391088 | 0.000230795 |
| Podel.06G106200 | Peonidin chloride | -0.890619154 | 0.000102352 |
| Podel.17G037900 | Peonidin chloride | -0.800803737 | 0.001748855 |
| Podel.07G028800 | Peonidin chloride | -0.806398917 | 0.001532005 |
| Podel.09G121500 | Peonidin chloride | -0.957129723 | 1.06107E-06 |
| Podel.02G205100 | Peonidin chloride | -0.94003742 | 5.51756E-06 |
| Podel.14G127200 | Ferulylpelargonidin di-O-hexosyl-O-pentoside | -0.861352785 | 0.000318212 |
| Podel.08G221200 | Cyanidin 3-O-glucosyl-malonylglucoside | -0.831661755 | 0.000796638 |
| Podel.14G127200 | Cyanidin 3-O-glucosyl-malonylglucoside | -0.816686037 | 0.001187721 |
| Podel.09G121500 | Cyanidin O-rutinoside | -0.873881127 | 0.000202585 |
| Podel.02G040700 | Petunidin 3-O-rutinoside | -0.882767997 | 0.000142785 |
| Podel.09G121500 | Cyanidin 3-O-glucoside | -0.836480275 | 0.000694894 |
| Podel.09G121500 | Delphinidin 3-sophoroside-5-rhamnoside | -0.847875249 | 0.000494126 |
| Podel.09G121500 | Cyanin chloride | -0.827801199 | 0.000886158 |
| Podel.06G106200 | Peonidin O-hexoside | -0.813888967 | 0.001274739 |
| Podel.09G121500 | Peonidin O-hexoside | -0.928346687 | 1.3178E-05 |
| Podel.02G205100 | Peonidin O-hexoside | -0.883383866 | 0.000139223 |
| Podel.09G121500 | Keracyanin chloride | -0.901334344 | 6.22719E-05 |
| Podel.09G121500 | Malvidin 3-galactoside chloride | -0.84536024 | 0.000533964 |
| Procyanidin A3 | Podel.06G106200 | -0.820281573 | 0.001082664 |
| Procyanidin A1 | Podel.06G106200 | -0.843186287 | 0.000570367 |
| Procyanidin A2 | Podel.06G106200 | -0.820417853 | 0.001078828 |
| Procyanidin A3 | Podel.09G121500 | -0.905507765 | 5.05334E-05 |
| Procyanidin A1 | Podel.09G121500 | -0.92722248 | 1.42173E-05 |
| Procyanidin B3 | Podel.09G121500 | -0.882617502 | 0.000143666 |
| Procyanidin A2 | Podel.09G121500 | -0.904497731 | 5.31995E-05 |
| cyanin chloride | Podel.09G121500 | -0.827801199 | 0.000886158 |
| Procyanidin B2 | Podel.09G121500 | -0.85267661 | 0.000424503 |
| Procyanidin A3 | Podel.06G234300 | -0.888202845 | 0.000113688 |
| Procyanidin A1 | Podel.06G234300 | -0.912016461 | 3.57672E-05 |
| Procyanidin B3 | Podel.06G234300 | -0.916848148 | 2.71927E-05 |
| Procyanidin A2 | Podel.06G234300 | -0.922999745 | 1.87131E-05 |
| cyanin chloride | Podel.06G234300 | -0.884980818 | 0.00013031 |
| Procyanidin B2 | Podel.06G234300 | -0.898579524 | 7.11243E-05 |
| Pseudopurpurin | Podel.06G234300 | -0.833229563 | 0.000762349 |
| Procyanidin A3 | Podel.02G205100 | -0.857546003 | 0.00036193 |
| Procyanidin A1 | Podel.02G205100 | -0.878122435 | 0.000172007 |
| Procyanidin A2 | Podel.02G205100 | -0.832798359 | 0.000771663 |
| Podel.04G021100 | Ferulylpelargonidin di-O-hexosyl-O-pentoside | -0.822059932 | 0.000589969 |
| Podel.04G021100 | Cyanidin 3-O-glucosyl-malonylglucoside | -0.812383794 | 0.000214337 |
| Podel.04G021100 | Cyanidin 3-O-malonylhexoside | 0.80243987 | 9.88923E-06 |
| Podel.04G021100 | Cyanidin O-acetylhexoside | 0.927677836 | 1.37888E-05 |
| Podel.04G021100 | Pelargonidin 3-O-malonylhexoside | 0.982601152 | 1.2196E-08 |
| Podel.04G021100 | Pelargonidin O-acetylhexoside | 0.848797734 | 0.000480107 |
| Podel.04G021100 | Peonidin chloride | 0.820138061 | 2.23485E-05 |
| Podel.04G021100 | Cyanidin O-rutinoside | 0.825848554 | 7.67844E-06 |
| Podel.04G021100 | Cyanidin 3-O-glucoside | 0.807476987 | 0.000117279 |
| Podel.04G021100 | Delphinidin 3-sophoroside-5-rhamnoside | 0.803300739 | 5.6501E-05 |
| Podel.04G021100 | Cyanin chloride | 0.814980818 | 0.00013031 |
| Podel.04G021100 | Peonidin O-hexoside | 0.810292692 | 9.23701E-05 |
| Podel.04G021100 | Keracyanin chloride | 0.862482383 | 5.49636E-07 |
| Podel.04G021100 | Malvidin 3-galactoside chloride | 0.800017397 | 0.001344869 |
| Podel.06G234300 | Cyanidin 3-O-malonylhexoside | -0.89525044 | 8.3108E-05 |
| Podel.06G234300 | Cyanidin O-acetylhexoside | -0.90010798 | 6.60988E-05 |
| Podel.06G234300 | Pelargonidin O-acetylhexoside | -0.804860241 | 5.22302E-05 |
| Podel.06G234300 | Peonidin chloride | -0.875020627 | 0.000193984 |
| Podel.06G234300 | Peonidin O-hexoside | -0.818914583 | 2.4063E-05 |
| Podel.06G234300 | Malvidin 3-galactoside chloride | -0.825609716 | 0.00018965 |
