# Supplementary material for: Transcriptome and metabolome analyses reveal molecular mechanisms of anthocyanin-related leaf color variation in poplar (Populus deltoides) cultivars
Source: Front Plant Sci. 2023 Feb 24;14:1103468. doi: 10.3389/fpls.2023.1103468 (PMC9998943; doi:10.3389/fpls.2023.1103468)
Supplement: Supplementary file 15 [file Table_14.docx]

**Supplementary Table 14 |** Connection network between bHLH TFs and anthocyanin metabolites.

| Var1 | Var2 | Cor | p_value |
| --- | --- | --- | --- |
| Podel.15G110700 | Cyanidin 3-O-glucosyl-malonylglucoside | -0.941955115 | 4.70528E-06 |
| Podel.01G109500 | Cyanidin 3-O-glucosyl-malonylglucoside | -0.970585817 | 1.65053E-07 |
| Podel.01G109500 | Delphinidin O-malonylhexoside | -0.835149551 | 0.000721924 |
| Podel.T132400 | Malvidin 3-galactoside chloride | -0.843830738 | 0.000559381 |
| Podel.T132400 | Cyanidin 3-O-glucoside | -0.826713398 | 0.000912732 |
| Podel.05G135800 | Cyanidin 3-O-glucoside | -0.835595362 | 0.000712779 |
| Podel.04G160100 | Pelargonidin 3-O-malonylhexoside | -0.816628232 | 0.001189472 |
| Podel.T132400 | Pelargonidin 3-O-malonylhexoside | -0.966824764 | 2.99333E-07 |
| Podel.05G135800 | Pelargonidin 3-O-malonylhexoside | -0.965197782 | 3.79251E-07 |
| Podel.14G157400 | Pelargonidin 3-O-malonylhexoside | -0.863032876 | 0.000300279 |
| Podel.09G122300 | Pelargonidin 3-O-malonylhexoside | -0.889174291 | 0.000109018 |
| Podel.15G110700 | Malvidin O-hexoside | -0.802739784 | 0.001671321 |
| Podel.T132400 | Cyanidin O-rutinoside | -0.87240935 | 0.000214132 |
| Podel.05G135800 | Cyanidin O-rutinoside | -0.85953853 | 0.000338502 |
| Podel.T132400 | Cyanidin O-acetylhexoside | -0.940934796 | 5.12471E-06 |
| Podel.05G135800 | Cyanidin O-acetylhexoside | -0.934621687 | 8.42362E-06 |
| Podel.14G157400 | Cyanidin O-acetylhexoside | -0.872407177 | 0.00021415 |
| Podel.09G122300 | Cyanidin O-acetylhexoside | -0.865929413 | 0.00027122 |
| Podel.T132400 | Pelargonidin O-acetylhexoside | -0.854726012 | 0.00039722 |
| Podel.05G135800 | Pelargonidin O-acetylhexoside | -0.810213318 | 0.001396452 |
| Podel.04G160100 | Cyanidin 3-O-malonylhexoside | -0.838093341 | 0.000663186 |
| Podel.T132400 | Cyanidin 3-O-malonylhexoside | -0.942857194 | 4.35754E-06 |
| Podel.05G135800 | Cyanidin 3-O-malonylhexoside | -0.954743341 | 1.38554E-06 |
| Podel.14G157400 | Cyanidin 3-O-malonylhexoside | -0.887755714 | 0.00011589 |
| Podel.09G122300 | Cyanidin 3-O-malonylhexoside | -0.900932527 | 6.35061E-05 |
| Podel.07G028600 | Peonidin chloride | -0.811328939 | 0.001358605 |
| Podel.04G160100 | Peonidin chloride | -0.848306765 | 0.000487529 |
| Podel.T132400 | Peonidin chloride | -0.938312928 | 6.33926E-06 |
| Podel.05G135800 | Peonidin chloride | -0.944353498 | 3.82586E-06 |
| Podel.14G157400 | Peonidin chloride | -0.890599316 | 0.000102441 |
| Podel.09G122300 | Peonidin chloride | -0.899066775 | 6.94907E-05 |
| Podel.T132400 | Delphinidin 3-sophoroside-5-rhamnoside | -0.847336059 | 0.000502466 |
| Podel.05G135800 | Delphinidin 3-sophoroside-5-rhamnoside | -0.845265304 | 0.000535515 |
| Podel.T132400 | Peonidin O-hexoside | -0.911148889 | 3.75096E-05 |
| Podel.05G135800 | Peonidin O-hexoside | -0.889967911 | 0.000105316 |
| Podel.14G157400 | Peonidin O-hexoside | -0.843177337 | 0.000570521 |
| Podel.09G122300 | Peonidin O-hexoside | -0.813113084 | 0.001299724 |
| Podel.T132400 | Keracyanin chloride | -0.90868566 | 4.2823E-05 |
| Podel.05G135800 | Keracyanin chloride | -0.875492438 | 0.000190507 |
| Procyanidin A3 | Podel.T132400 | -0.865431069 | 0.000276056 |
| Procyanidin A2 | Podel.T132400 | -0.882101462 | 0.000146719 |
| Procyanidin A1 | Podel.T132400 | -0.895833467 | 8.09026E-05 |
| cyanin chloride | Podel.T132400 | -0.828000066 | 0.000881365 |
| Procyanidin B3 | Podel.T132400 | -0.863052061 | 0.000300079 |
| Procyanidin A3 | Podel.05G135800 | -0.891825401 | 9.70343E-05 |
| Procyanidin A2 | Podel.05G135800 | -0.895204912 | 8.32822E-05 |
| Procyanidin A1 | Podel.05G135800 | -0.910558431 | 3.87331E-05 |
| cyanin chloride | Podel.05G135800 | -0.814784492 | 0.001246363 |
| Procyanidin B3 | Podel.05G135800 | -0.877144663 | 0.000178713 |
| Procyanidin A3 | Podel.14G157400 | -0.805955353 | 0.001548401 |
| Procyanidin A1 | Podel.14G157400 | -0.836111414 | 0.000702306 |
| Procyanidin A3 | Podel.09G122300 | -0.827025918 | 0.000905035 |
| Procyanidin A2 | Podel.09G122300 | -0.819073393 | 0.001117132 |
| Procyanidin A1 | Podel.09G122300 | -0.846283554 | 0.00051906 |
| Podel.02G199200 | Cyanidin 3-O-glucosyl-malonylglucoside | 0.914084418 | 3.18702E-05 |
| Podel.03G099000 | Cyanidin 3-O-glucosyl-malonylglucoside | 0.938753263 | 6.12081E-06 |
| Podel.07G028600 | Cyanidin 3-O-glucosyl-malonylglucoside | 0.961452661 | 6.28224E-07 |
| Podel.04G160100 | Cyanidin 3-O-glucosyl-malonylglucoside | 0.947666448 | 2.83067E-06 |
| Podel.T132400 | Cyanidin 3-O-glucosyl-malonylglucoside | 0.884232305 | 0.00013443 |
| Podel.05G135800 | Cyanidin 3-O-glucosyl-malonylglucoside | 0.962769584 | 5.29175E-07 |
| Podel.14G157400 | Cyanidin 3-O-glucosyl-malonylglucoside | 0.91091694 | 3.79865E-05 |
| Podel.09G122300 | Cyanidin 3-O-glucosyl-malonylglucoside | 0.984334183 | 7.2384E-09 |
| Podel.02G199200 | Delphinidin O-malonylhexoside | 0.942335247 | 4.55616E-06 |
| Podel.03G099000 | Delphinidin O-malonylhexoside | 0.919947511 | 2.2609E-05 |
| Podel.07G028600 | Delphinidin O-malonylhexoside | 0.913410278 | 3.31019E-05 |
| Podel.04G160100 | Delphinidin O-malonylhexoside | 0.89965696 | 6.75518E-05 |
| Podel.09G122300 | Delphinidin O-malonylhexoside | 0.829809253 | 0.000838676 |
| Podel.01G333900 | Myrtillin chloride | 0.935627143 | 7.80891E-06 |
| Podel.15G110700 | Myrtillin chloride | 0.840496952 | 0.000618029 |
| Podel.02G199200 | Pelargonin chloride | 0.877383022 | 0.00017706 |
| Podel.03G099000 | Pelargonin chloride | 0.846196929 | 0.000520444 |
| Podel.01G333900 | Cyanidin 3-O-glucoside | 0.938879497 | 6.0593E-06 |
| Podel.15G110700 | Cyanidin 3-O-glucoside | 0.934881258 | 8.26139E-06 |
| Podel.01G333900 | Pelargonidin 3-O-malonylhexoside | 0.807260572 | 0.001500537 |
| Podel.15G110700 | Pelargonidin 3-O-malonylhexoside | 0.966250676 | 3.25829E-07 |
| Podel.01G109500 | Pelargonidin 3-O-malonylhexoside | 0.859844822 | 0.000335007 |
| Podel.02G199200 | Petunidin-3-O-glucoside chloride | 0.890479704 | 0.000102981 |
| Podel.03G099000 | Petunidin-3-O-glucoside chloride | 0.857117956 | 0.000367123 |
| Podel.07G028600 | Petunidin-3-O-glucoside chloride | 0.86220759 | 0.000308987 |
| Podel.04G160100 | Petunidin-3-O-glucoside chloride | 0.823540867 | 0.000993757 |
| Podel.01G333900 | Idaein chloride | 0.934538421 | 8.47619E-06 |
| Podel.15G110700 | Idaein chloride | 0.850517539 | 0.000454797 |
| Podel.02G199200 | Petunidin 3-O-rutinoside | 0.841783516 | 0.000594858 |
| Podel.03G099000 | Petunidin 3-O-rutinoside | 0.817448096 | 0.001164826 |
| Podel.01G333900 | Cyanidin O-rutinoside | 0.925230331 | 1.62171E-05 |
| Podel.15G110700 | Cyanidin O-rutinoside | 0.952520407 | 1.7543E-06 |
| Podel.01G333900 | Cyanidin O-diacetyl-hexoside-O-glyceric acid | 0.922449841 | 1.93726E-05 |
| Podel.15G110700 | Cyanidin O-diacetyl-hexoside-O-glyceric acid | 0.849536647 | 0.000469102 |
| Podel.15G110700 | Cyanidin O-acetylhexoside | 0.866279588 | 0.000267861 |
| Podel.01G109500 | Cyanidin O-acetylhexoside | 0.832275952 | 0.000783066 |
| Podel.01G333900 | Cyanidin O-malonyl-malonylhexoside | 0.86727407 | 0.000258498 |
| Podel.01G333900 | Cyanidin O-syringic acid | 0.933455123 | 9.18411E-06 |
| Podel.15G110700 | Cyanidin O-syringic acid | 0.846301822 | 0.000518768 |
| Podel.15G110700 | Cyanidin 3-O-malonylhexoside | 0.88982714 | 0.000105965 |
| Podel.01G109500 | Cyanidin 3-O-malonylhexoside | 0.866096148 | 0.000269616 |
| Podel.15G110700 | Peonidin chloride | 0.889079426 | 0.000109468 |
| Podel.01G109500 | Peonidin chloride | 0.866407047 | 0.000266646 |
| Podel.01G333900 | Delphinidin 3-sophoroside-5-rhamnoside | 0.946321884 | 3.20613E-06 |
| Podel.15G110700 | Delphinidin 3-sophoroside-5-rhamnoside | 0.954184657 | 1.47181E-06 |
| Podel.15G110700 | Peonidin O-hexoside | 0.806093288 | 0.001543288 |
| Podel.01G333900 | Keracyanin chloride | 0.938305078 | 6.34321E-06 |
| Podel.15G110700 | Keracyanin chloride | 0.962579613 | 5.4264E-07 |
| Pseudopurpurin | Podel.01G333900 | 0.960792535 | 6.83127E-07 |
| cyanin chloride | Podel.01G333900 | 0.963870781 | 4.56258E-07 |
| Procyanidin B3 | Podel.01G333900 | 0.910766472 | 3.82985E-05 |
| Procyanidin A3 | Podel.15G110700 | 0.852175005 | 0.000431397 |
| Pseudopurpurin | Podel.15G110700 | 0.897104709 | 7.62529E-05 |
| Procyanidin A2 | Podel.15G110700 | 0.924249737 | 1.72797E-05 |
| Procyanidin A1 | Podel.15G110700 | 0.871812621 | 0.000218958 |
| cyanin chloride | Podel.15G110700 | 0.932188158 | 1.00705E-05 |
| Procyanidin B3 | Podel.15G110700 | 0.946607523 | 3.12325E-06 |
| Procyanidin A3 | Podel.01G109500 | 0.804535822 | 0.001601777 |
| Procyanidin A2 | Podel.01G109500 | 0.812469579 | 0.00132073 |
| Procyanidin A1 | Podel.01G109500 | 0.834343981 | 0.000738678 |
